# Progressive promoter element combinations classify conserved orthogonal plant circadian gene expression modules

**DOI:** 10.1098/rsif.2014.0535

**Published:** 2014-10-06

**Authors:** Sandra P. Smieszek, Haixuan Yang, Alberto Paccanaro, Paul F. Devlin

**Affiliations:** 1 School of Biological Sciences, Royal Holloway University of London, Egham TW20 0EX, UK; 2 Centre for Systems and Synthetic Biology, Royal Holloway University of London, Egham TW20 0EX, UK; 3 Department of Computer Science, Royal Holloway University of London, Egham TW20 0EX, UK

**Keywords:** clock, dimensionality reduction, microarray, phase, Random Forest

## Abstract

We aimed to test the proposal that progressive combinations of multiple promoter elements acting in concert may be responsible for the full range of phases observed in plant circadian output genes. In order to allow reliable selection of informative phase groupings of genes for our purpose, intrinsic cyclic patterns of expression were identified using a novel, non-biased method for the identification of circadian genes. Our non-biased approach identified two dominant, inherent orthogonal circadian trends underlying publicly available microarray data from plants maintained under constant conditions. Furthermore, these trends were highly conserved across several plant species. Four phase-specific modules of circadian genes were generated by projection onto these trends and, in order to identify potential combinatorial promoter elements that might classify genes into these groups, we used a Random Forest pipeline which merged data from multiple decision trees to look for the presence of element combinations. We identified a number of regulatory motifs which aggregated into coherent clusters capable of predicting the inclusion of genes within each phase module with very high fidelity and these motif combinations changed in a consistent, progressive manner from one phase module group to the next, providing strong support for our hypothesis.

## 1. Introduction

Circadian clocks are ubiquitous and are found in bacteria, fungi, plants and animals [1]. They constitute endogenous 24 h systems that allow organisms to anticipate changes in the daily environment. In plants, the circadian clock can be directly linked to yield potential through adaptive advantage and hybrid vigour [2,3]. The clock plays a pervasive role in temporal compartmentalization of diurnal and photoperiodic responses, including photosynthetic capacity, floral transition, photomorphogenesis and stress responses and, hence, contributes to proper growth and development [4].

At the heart of the clock in higher organisms is a central transcriptional feedback loop. The model of the *Arabidopsis thaliana* circadian clock consists of a central loop termed a repressilator, with three groups of clock proteins, each repressing expression of the previous one in turn to form a complete loop which oscillates with a 24 h period [5]. However, it is not well understood how the circadian ‘output genes’ are regulated by this central clock mechanism in plants.

One way to infer the topology of clock transcriptional regulation is to construct a network that relies upon regulatory elements residing in the promoters. A small number of individual circadian *cis* elements have been identified in plants by looking for enrichment of a particular sequence among the promoters of genes sharing a common timing or phase [6]. However, recent studies have shown that it is unlikely that the rhythmicity of clock-regulated genes is induced via the action of regulatory proteins on a single *cis* element sequence [7]. For example, in *Arabidopsis*, circadian regulation of *ELF4* is exerted via positive action of the transcription factors FHY3, FAR1 and HY5, acting through fbs and ACE *cis* elements; and negative regulation by the transcription factors CCA1 and LHY, acting through the evening element [8].

The identification of circadian genes is, of course, the critical first step for in-depth understanding of the network topology of clock regulation. Much microarray data are publicly available from plant circadian time courses and a range of approaches have been used to identify circadian genes. Identification of circadian genes varies greatly from one method to another and no defined subset of plant circadian genes has been agreed upon [9]. Existing approaches commonly involve supervised selection of genes fitting to certain predefined patterns. However, such approaches are, by definition, biased. In order to form phase groupings of output genes which genuinely reflect the action of distinct driving transcription actors, a non-biased method is required. Despite the undisputed utility of Fourier theory as a non-biased method for the identification of rhythmic patterns in time series, there are limitations to the applicability of this method for short time series with a low resolution such as those that have been generated by microarray analyses.

In this study, we proposed that global patterns of circadian output gene expression may be explained by the concerted action of multiple promoter elements within each gene, and that the element combinations driving expression of successive groups of genes change gradually in a progressive manner. We have used a machine-learning, decision-tree-based approach, Random Forest (RF), to go beyond the established single *cis* element analysis approaches and search for combinations of *cis* elements which, in concert, classify *Arabidopsis* circadian genes into phase-specific modules. With a view to identifying the inherent phase modules of circadian genes, we developed a linear projection method as a non-biased method of identifying trends underlying short time-course circadian microarray data. Circadian pathways have been shown to be conserved across several plant species [10], and so this approach was also applied across species to examine conservation of the trends. Comparison of inferred *cis* element combinations from each phase module demonstrated that progressive patterns of element combinations do determine the phase of *Arabidopsis* circadian output genes.

## 2. Material and methods

### 2.1. Datasets

The following transcriptomic datasets were used in this study: *A. thaliana* (Affymetrix Arabidopsis ATH1 Genome Array): GSE8365, GSE5612; *Zea mays* (105 K Agilent Microarray): GSE23918 GSE31763; *Oryza sativa* (57 K Affymetrix Rice Whole Genome Array): GSE28124. Orthologous genes were identified using the integrative orthology method available from PLAZA and BIOMART, and the upstream promoter sequences were retrieved from RSAT.

### 2.2. Pre-processing

Prior to application of dimensionality reduction, data were pre-processed. The microarray gene expression data are represented as an ***n*** × ***m*** matrix ***X*** with ***n*** genes (rows) under ***m*** conditions (columns). The pre-processing procedure [11] involved log_2_ transformation, centring of the columns by subtracting the average, column normalization, centring the rows by subtracting the average and then row normalization. From the resultant data, a covariation matrix was constructed and then independent component analysis (ICA) was performed. The resulting gene transcriptional responses had a mean of 0 and unit standard deviation.

### 2.3. Gene regulation by independent component analysis

The FastICA package for Matlab (http://research.ics.aalto.fi/ica/fastica/) was used to carry out ICA on the microarray dataset. As the FastICA algorithm relies on random initializations for its maximization and faces the problem of convergence to local optima, we iterated FastICA 100 times and took the average in order to alleviate the instability of the slightly different results in each iteration. ICA measures the interestingness of a linear combination *a^T^x* in terms of the size of its absolute kurtosis. After pre-processing and normalization, the ICA model for gene expression data can be expressed as: *X* = *AS*. In this ICA model, the columns of 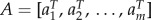 are the *n* × *m* latent vectors of the gene microarray data. Each column of *A* is associated with a specific gene expression mode. *S* contains the *m* × *m* gene signatures where the rows of *S* are statistically independent of each other. The gene profiles in *X* are considered to be a linear mixture of statistically independent components *S* combined by an unknown mixing matrix A. Once latent variable matrix ***A*** was obtained, the corresponding elementary modes were identified to extract information for classification.

### 2.4. Empirical significance test for independent component analysis

To test whether *d* independent components are significant in representing the whole microarray data, we designed an empirical significance test. The idea is that, if the data matrix X can be properly represented by *d* independent components, then the reconstruction error 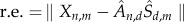 should be small, where 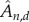 and 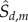 are estimated by an ICA algorithm, and ||·|| denote the Euclidean distance between *X* and the reconstruction 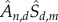. As a result, randomized reconstruction errors, which are obtained by randomizing *X*, learning *A* and *S*, and calculating the reconstruction error, have a probability to be larger than r.e. Based on this idea, we report an empirical *p*-value to test how much *d* independent components are significant in the ICA by the following procedure. For the original data *X*, we ran ICA, and obtained r.e*.* We then randomized *X* to *RX* by a rotations-based procedure SwapDiscretized [12], which guarantees that the distributions of the discretized values in the rows and columns do not change. For RX, we ran the ICA procedure to obtain a randomized r.e. (r.r.e.). We then repeated steps 2 and 3 1000 times so that we had 1000 r.r.e. values. Finally, the empirical *p*-value was the frequency with which r.r.e. values are smaller than r.e.

### 2.5. Projection down to a subspace and goodness of embedding

Projection scatter plot coordinates *q_i_*_,*k*_ for transcriptional response *n_i_* projected on component *v_k_* were calculated as *q_i_*_,*k*_ = *n_i_* · *v_k_*. The gene transcriptional responses were projected onto the two ICA-derived circadian components described in the Results section. A stringent radial cut-off of 0.8 was used following projection in order to define circadian genes, this being a commonly used cut-off for Pearson analyses [13]. The genes were later sorted with regards to their angular positions.

### 2.6. Module detection and preservation

Genes classified as circadian were divided into modules based on the eigengene axis to which they showed highest dot product score. Two types of analysis were performed to assess the preservation of patterns between datasets. One involved assessment of similarity of eigengenes across datasets; the second involved assessment of conservation of circadian orthologues in terms of transcriptomic profiles. For analysis of similarity of eigengenes across datasets, concurrence was confirmed by Pearson correlation analysis. The integrative correlation coefficient [14] was used as a measure of cross-study reproducibility for gene expression array data. For this, we compare two microarray studies, *S_a_* and *S_b_*, with sample size of *n_a_* and *n_b_*, respectively, and a total of *m* common genes. All genes were transformed to the same distribution, that is, mean zero and variance one. The expression vector for a gene *x* in study *S_a_* is notated as *x_a_. A* denotes the (*m* − 1) × *n_a_* data matrix for study *S_a_* without gene *x*. Similarly, *B* denotes the (*m* − 1) × *n_b_* data matrix for study *S_b_* without gene *y*. Furthermore, *cE_m_* is the *m* × *m* matrix with every element equal to *c* and *I_m_* denotes the *m* × *m* identity matrix. If *x* and *y* are two random vectors of length *m*, then 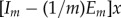 returns 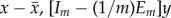 returns 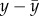 and cov(*x*,*y*) = *y^t^*[*I_m_* − (1/*m*)*E_m_*]^2^*x*. Denote *I_m_* = [*I_m_* − (1/*m*)*E_m_*]^2^. The integrative correlation coefficient for gene *x* in studies *S_a_* and *y* in *S_b_* can be defined as
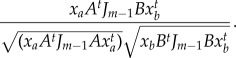


### 2.7. Jaccard index for modules

We used the Jaccard similarity score TP/(TP + FP + FN) to asses module composition [15], where TP is true positive, FP is false positive and FN is false negative. The similarity of two modules is measured by the Jaccard index score between the edges of two co-expression graphs whose nodes are the members of the modules and whose edges are those pairs with a co-expression greater than or equal to 0.8. This involves permutation to derive significance scores.

### 2.8. Classification based on promoter elements

To assess the ability of combinations of promoter elements to correctly predict the phase module class of our circadian genes, an RF approach was used. One-kilobase-pair lengths of promoter sequences were selected and matrices of individual promoter elements were constructed following a simple over-representation analysis based on all combinations of 5–8 mers of letters (A,T,C,G). The 21 most overrepresented individual features from this analysis were then selected for combinatorial analysis by RF (electronic supplementary material, table S1). Classification was performed using Random Forest Matlab (http://code.google.com/p/randomforest-matlab). This was portrayed by a NetLOGO model (http://ccl.northwestern.edu/netlogo). Trees were grown from different randomly selected subsets of four features from among the 21 possible (this being the standard ‘mtry’, equal to the integer value of the square root of the number of possible features). To measure performance, two-thirds cross validation was used. In RF, a tree or classifier is selected if it performs well according to internally defined criteria in cross validation between the training and the remaining samples. Training samples were randomly selected, with the training set consisting of two-thirds of the samples, including an equal number of genes from each of the phase classes. The selected scheme was an RF with 2000 trees. Final classification is made by combining results from trees via voting. The relative contribution (importance) of each promoter motif (variable) to the classification of the genes is given in terms of a variable importance (VIMP) measure.

The performance of RF in correctly classifying genes within each phase module was assessed using receiver operating characteristic (ROC) curves.

## 3. Results

### 3.1. Pattern inference through independent component analysis

In order to test our hypothesis that progressive combinations of promoter elements acting in concert allow the realization of the full range of possible phases of circadian output genes, we carried out a combinatorial analysis of potential promoter elements determining phase of expression. We began by developing an unbiased method to identify circadian patterns of gene expression from within relatively short timecourse transcriptomic data. We argued that this marks an improvement for this particular purpose on the current favoured method of looking for preconceived patterns of gene expression as it would allow identification of natural groupings of output genes which may reflect regulation by common transcription factor combinations. We employed ICA to identify dominant descriptive components or trends, which we have termed ‘eigentrends’, within data. This was followed by projection of all data onto these dominant eigentrends. Our hypothesis was that, in the absence of variation in external stimuli, variation in gene expression over a multi-day, free-running time course would be primarily due to circadian processes within the organism. We proposed that these circadian patterns should, therefore, constitute the eigentrends which account for the most variation within the data.

A classical component analysis technique for detecting and visualizing relevant information from measured data is principal component analysis (PCA). However, the fact that PCA necessarily identifies orthogonal components (uncorrelated with the preceding components) is problematic. In looking for circadian patterns of expression, we wished to remove this restriction as only two cycling orthogonal components with the same period are mathematically possible (essentially, sine and cosine patterns). Instead, we used ICA, a variation on PCA which does not impose the limit of orthogonality [11].

**Figure 1.**
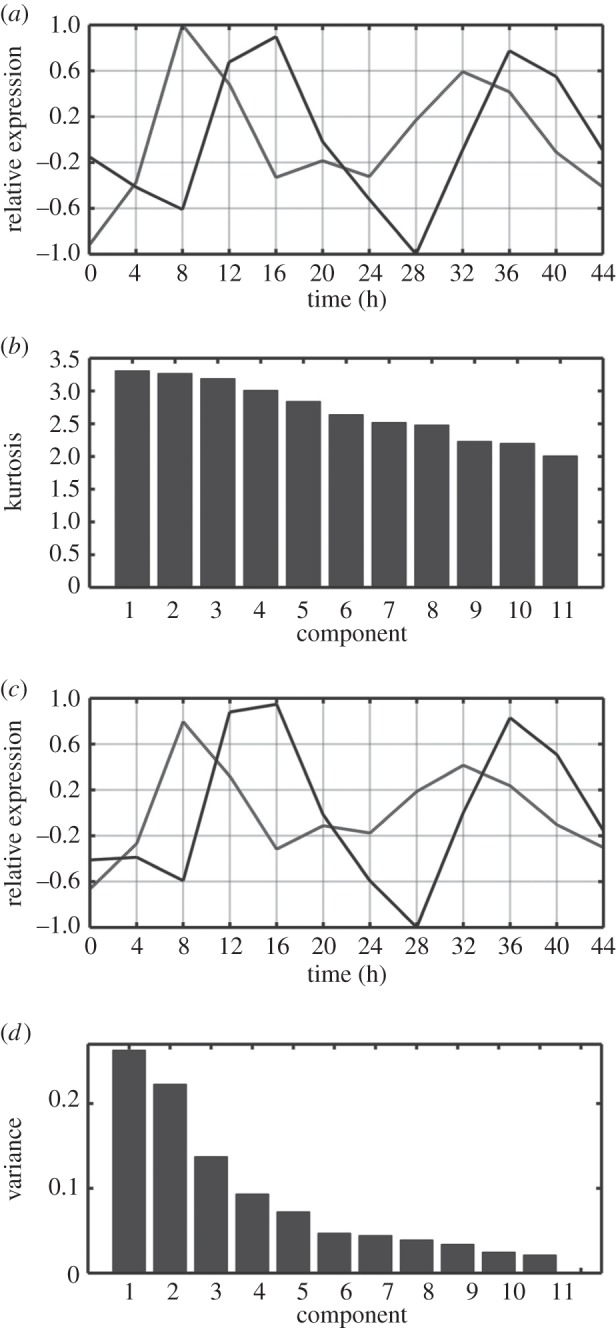
ICA and PCA performed on *Arabidopsis* circadian microarray time series. (*a*) First (peak at 8 h) and second (peak at 12–16 h) independent components plotted against time since subjective dawn. (*a*) The kurtosis of a frequency distribution of data projected along each independent component. (*c*) First (peak at 8 h) and second (peak at 12–16 h) principal components. (*d*) Variance within the data captured by each principal component.

We began by analysing a well-characterized microarray dataset from *Arabidopsis* [16]. Plants had been entrained in light/dark cycles prior to release into constant light. The dataset comprised approximately 22 810 transcripts monitored for 12 time points taken at 4 h intervals. For practical reasons, we used the degree of kurtosis to sort the eigentrends [17]. We reasoned that latent trends with the most negative kurtosis can give us the most relevant information on the basis that more data points than expected will be positively or negatively correlated with these eigentrends. After ordering the eigentrends by kurtosis, the two most significant eigentrends were apparently circadian, supporting this proposal, whereas the third possibly represents a damping rhythm (figure 1*a,b*; electronic supplementary material, figure S1): a rhythm gradually lost following transfer to constant conditions. Subsequent eigentrends beyond the third showed an apparently random pattern.

**Figure 2.**
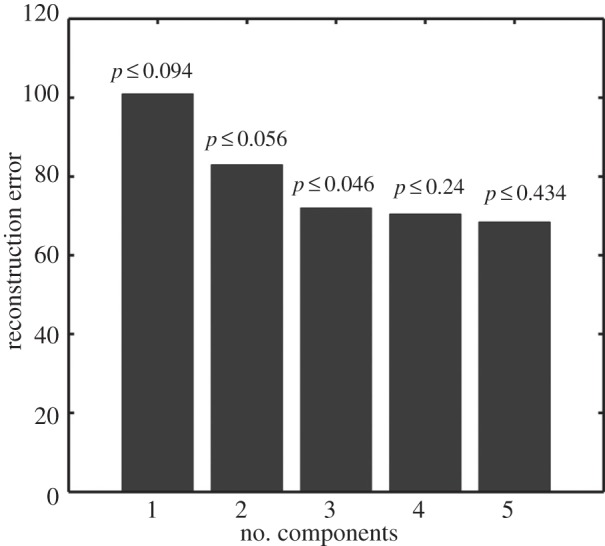
Reconstruction error for ICA components of an *Arabidopsis* circadian microarray time series. After ordering by kurtosis, the variance reconstruction error was plotted for addition of successive independent components. *p*-values are shown for an empirical significance test to determine the number of independent components which are significant in terms of representing the whole microarray data.

Following an ordering of eigentrends by kurtosis, we determined how many were truly significant in terms of representing the data. The data were projected onto a subspace defined by the eigentrends and then compared with the original data by measuring the distance between the original data and their estimates based on these components. This was done initially for the first eigentrend then recalculated as each subsequent eigentrend was added. We observed that the subsequent addition of eigentrends after the first three did not strongly decrease reconstruction error, suggesting that we have three significant eigentrends (figure 2). We also devised an empirical significance test to determine the number of eigentrends which are significant in terms of representing the whole microarray data. Here, ICA was carried out and reconstruction errors were calculated for 1000 datasets, where the expression values within each gene's expression pattern were randomized. For each possible number of components, we calculated the frequency that the randomized reconstruction errors were smaller than the calculated reconstruction error for the actual data. This approach also demonstrated that the first three eigentrends significantly described the data, while one, two, four or five eigentrends did not (figure 2). Thus, ICA led to delineation of three apparently biologically relevant trends. The first two of these are ‘circadiantrends’, with the third component probably representing a dampening rhythm. The two ‘circadiantrends’ delineated by ICA correspond to four phases (positive and negative impressions of the curves) having approximately 4/8 h difference.

On comparing the results with a PCA of the same data, we observed that the two apparently circadian trends identified by ICA are also remarkable in almost exactly concurring with the first two eigentrends identified by PCA (figure 1*c*,*d*). The concurrence was confirmed by correlation analysis, giving an *R*-value of 0.9987 and 0.9972 for the first and second eigentrends, respectively. This agreement between results of ICA and PCA indicates that the two circadian eigentrends identified by ICA are orthogonal despite this not being imposed by the method. We propose that this mathematical orthogonality could possibly represent an underlying biological orthogonality in the way that rhythms are generated at the molecular level.

### 3.2. Assignment of circadian genes through projection into two-dimensional subspace

**Figure 3.**
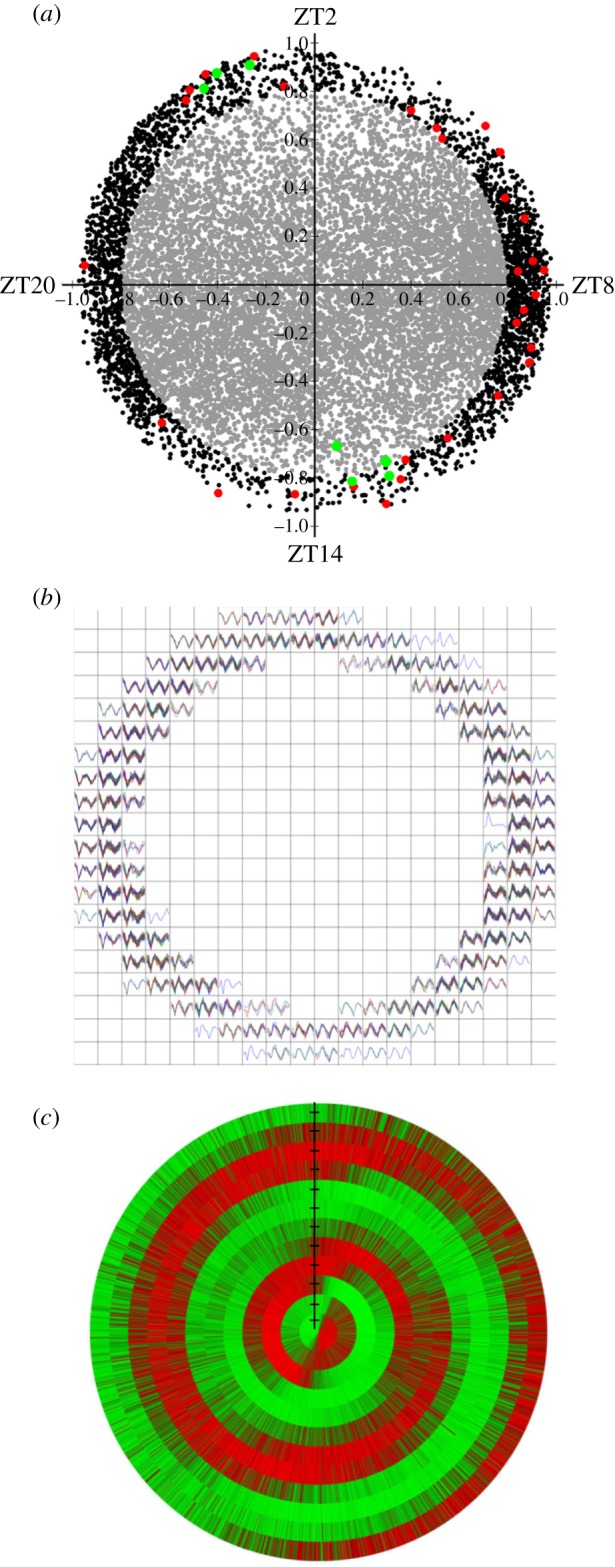
(*a*) Circadian genes identified by projection onto ICA components. Expression patterns of all genes from *Arabidopsis* circadian time-course data were projected onto the first (*x*-axis) and second (*y*-axis) ICA components. Black dots represent circadian genes, defined as lying at a distance of at least 0.8 from the origin. Grey dots represent those classed as non-circadian. Green dots represent the genes of the central clock loop (CCA1, LHY, TOC1, PRR9, ELF3, ELF4 and LUX). Red dots represent other oscillator-associated genes. Zt: Zeitgeber time. (*b*) Expression profiles of circadian genes. The projection scatter plot was divided into a 20 × 20 grid and all the genes in each grid box are displayed together as line graphs. (*c*) Circular heat map of gene expression in which angular positions of genes were used to order rows. Each row from the outside in represents the data points for a single gene across the circadian experiment. The circular map shows normalized gene expression patterns. Red, high expression; green, low expression.

Having identified two dominant circadian eigentrends and, therefore, four dominant oscillatory patterns, we sought to identify circadian genes themselves by sorting the data by similarity in expression to these eigentrends. This operated on the assumption that circadian genes will show strong dot product projection similarity to one or both of the two key circadian eigentrends that we identified and, thus, fall in a circular region some distance from the origin. This method was chosen in favour of a simple Pearson correlation. In a correlation scatter plot, the significance of genes with a low level of expression, whose pattern of expression might be considered as noise, can appear to be magnified. Effectively, the separation between signal and noise genes is decreased for a correlation compared with a projection scatter plot. We imposed a cut-off at a distance of 0.8. Using this approach, we classified 2948 genes as circadian (figure 3*a* and electronic supplementary material, table S2). Here, the radial coordinate constitutes a measure of cyclicity. This is depicted in figure 3*a* where the outermost genes are those we define as circadian genes. Effectively, the angular position around the plot represents the phase of expression.

As expected this method identified circadian genes defining the full range of possible phases. We noted a distinct clustering for genes showing high dot product with the first component on the *x*-axis (figure 3*a*). This would suggest that the most prevalent phase of peak expression is around midday. This subset was inspected for known core circadian genes and for all circadian genes identified as associated with the circadian clock according to The Arabidopsis Information Resource Gene Ontology (GO) database (those in the GO group, GO:0007623). All of these core genes examined were found to be present in the circadian subset (figure 3*a*, red dots). Core genes include *CIRCADIAN CLOCK-ASSOCIATED* (*CCA1*), *LATE ELONGATED HYPOCOTYL* (*LHY*), *PSEUDORESPONSE REGULATOR* (*PRR*) *3,* 7 and 9, *TIMING OF CAB EXPRESSION 1* (*TOC1*), *LUX ARRHYTHMO* (*LUX*) and *EARLY FLOWERING 3* (*ELF3*). Furthermore, *CCA1* and *LHY* were found to be in antiphase with *TOC1*, as indicated by the opposing positions in the agreement with their known antiphasic pattern of expression. We also noted that the core clock genes tended to be found directly close to the axis representing the second component, whereas the remaining population of circadian regulated genes were found spread around the circadian subspace with a clustering around the axis of the first component, approximately 6 h behind these effectors.

Our identification of 2965 circadian probe-sets or 2948 distinct circadian genes within the Covington dataset is comparable to the 2897 circadian probe-sets or 2885 distinct circadian genes identified by Covington *et al.* [16] using the regression-based COSOPT method [18]. However, our method identified only 1164 genes in common with the COSOPT method, highlighting the fact that this approach is quite distinct from the most commonly used biased approach to circadian microarray analysis. The identification of circadian genes is dependent on the method used and, given the range of methods available, no benchmark set of *Arabidopsis* circadian genes has been defined for comparison [9]. We present our method as an addition to the range of methods currently available. We do not claim that our circadian set provides more valid insights into the range of circadian processes ongoing in *Arabidopsis*. As stated, our aim in deriving this method was to group genes by phase on the basis of inherent patterns within the data with a view to improving our understanding of the action of regulatory motifs responsible for determination of phase. The genuine oscillatory pattern of our selected genes is demonstrated in figure 3*b,c*. Two approaches were used to demonstrate this. Firstly, the changing oscillatory patterns of the individual genes plotted around the projection are presented in figure 3*b*. Secondly, genes were ordered through angular positions in the ICA result around a circular heat map (figure 3*c*). These approaches also portray the inherent topology of the data as a continuous distribution. This further confirms the advantage of this method as a non-biased approach for the identification of circadian genes. Here, clustering, an alternative non-biased approach, would force cluster topology on a continuous distribution, preventing characterization of individual genes along such a continuous distribution. Our method, furthermore, adds the ability to order circadian genes by more than just peak phase, as has been used in the case of biased pattern-matching approaches. Genes sharing peaks but potentially having quite different patterns of expression in terms of peak shape could not be ordered solely on the basis of peak time, whereas our method also allows such additional information to be considered. Despite the fact that samples were taken only every 4 h, the shapes of the peaks clearly show a much greater range of patterns than the limited sign wave, square wave, triangular wave or regular spike patterns used in previous pattern-fitting methods and this additional information is used by our approach in ordering the genes.

### 3.3. Robustness of the circadian clock gene expression modules

**Figure 4.**
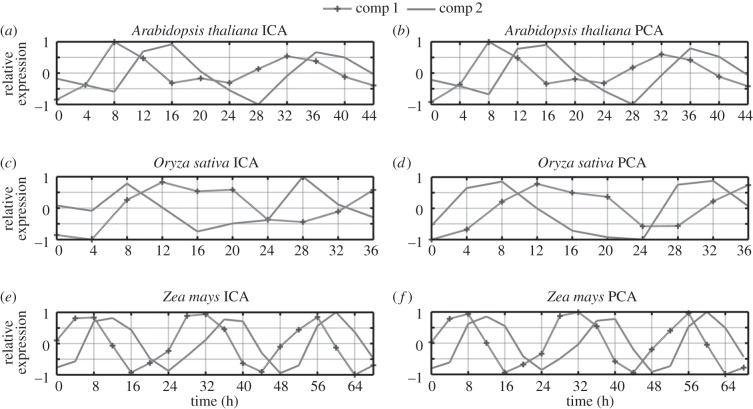
Comparative analysis of circadian time series. Dominant trends for circadian time-course transcriptomic data across species are portrayed through ICA (*a*,*c*,*e*) and PCA (*b*,*d*,*f*) plots. A second *Arabidopsis* transcriptomic dataset (*a,b*) and datasets for *O. sativa* (*c,d*) and *Z. mays* (*e,f*) were analysed.

We also applied this method to additional datasets. We chose a second dataset for *Arabidopsis* and additional datasets for *Z. mays*, and *O. sativa*, all generated using the same entraining and free-running conditions [19–21]. By repeating the discussed workflow, we found that the pattern of eigentrends was highly preserved in another dataset for *Arabidopsis* and in other species (figure 4). In all cases, there were two dominant eigentrends which were cyclic in nature with a periodicity close to 24 h. These same circadiantrends were identified using both ICA and PCA, and this finding suggests that the two orthogonal eigentrends found in the circadian data in the first *Arabidopsis* sample are conserved throughout a range of plants, encompassing both the monocots and dicots, further suggestive of an underlying biological significance that is also conserved. Strikingly, the position of key circadian orthologues was also preserved in projection scatter plots (data not shown). This is consistent with previous analysis which has shown that circadian orthologues correlate very well between different plant species and are, thus, expressed at approximately the same phase [22].

**Figure 5.**
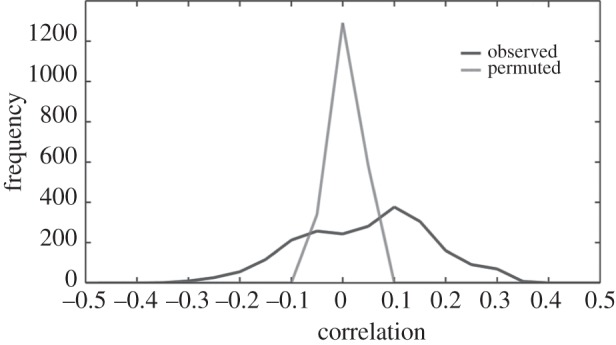
Integrative correlation coefficient between species. Distribution of the correlation coefficient between *Arabidopsis* and *Oryza sativa* orthologous circadian genes. The orthologous circadian genes show significantly higher integrative correlations using actual observed data (black line) than the null distribution, which was generated using permuted data where gene expression values within each gene were randomized (grey line).

We also confirmed conservation of the wider patterns themselves across species using a comparative mathematical framework. Orthologues were found between *O. sativa* and *Arabidopsis*. Altogether, 2209 orthologues were found for *O. sativa*. Cross-species microarray comparisons are complicated by noise, assignment of homology, probe quality, platform variations, laboratory effects, genetic background, dynamic environments and the status of the plant. However, we used the correlation coefficient as a measure to infer that the transcriptional behaviour of circadian genes is highly conserved across species. This metric assumes that, whereas the overall raw expression values may vary between studies, the intergene correlations will be more invariant [14]. We followed the method suggested by Doherty & Kay [9]. For all *Arabidopsis* circadian genes (GSE8365 dataset) with a circadian orthologue in rice, the correlation of each gene with its orthologue was determined, then a frequency distribution of these correlations was plotted (figure 5). The distribution of correlation values for all circadian probes shows that orthologous genes have a much higher correlation than a negative control representing correlation between orthologous genes when data points for each gene were randomly shuffled (figure 5). In the test data, 365 genes had corCor > 0.1; in the negative control no gene had corCor > 0.08. This clearly suggests that when we look at the data globally, taking the entire transcriptome into consideration, a large proportion of circadian orthologues are expressed in a conserved way.

The fact that individual gene patterns are conserved in addition to the conservation of the eigentrends suggests that the overall architecture of our circadian four ‘modules’ is also preserved. That further opened novel avenues for research into the evolution of circadian networks. The significance of this modularity was assessed across species datasets using the Jaccard similarity score [15]. The Jaccard coefficient measures similarity between sample sets (modules). It assesses the number of common members of two sets as a proportion of the total number of members in the two sets. This was used to score the overlap between two equivalent module compositions across *Arabidopsis* and rice. Additionally, randomizations were used to define the significance of each specific score. This produced a normalized similarity score for each module, expressed as the number of standard deviations from the mean of the distribution of Jaccard similarity scores for equivalent randomized module structures (electronic supplementary material, table S3). For the four phase module classes, the number of standard deviations from the mean was between 35 and 61, indicating an extremely significant conservation of the members of these four phase modules.

### 3.4. Identification of circadian promoter element combinations using Random Forest

**Figure 6.**
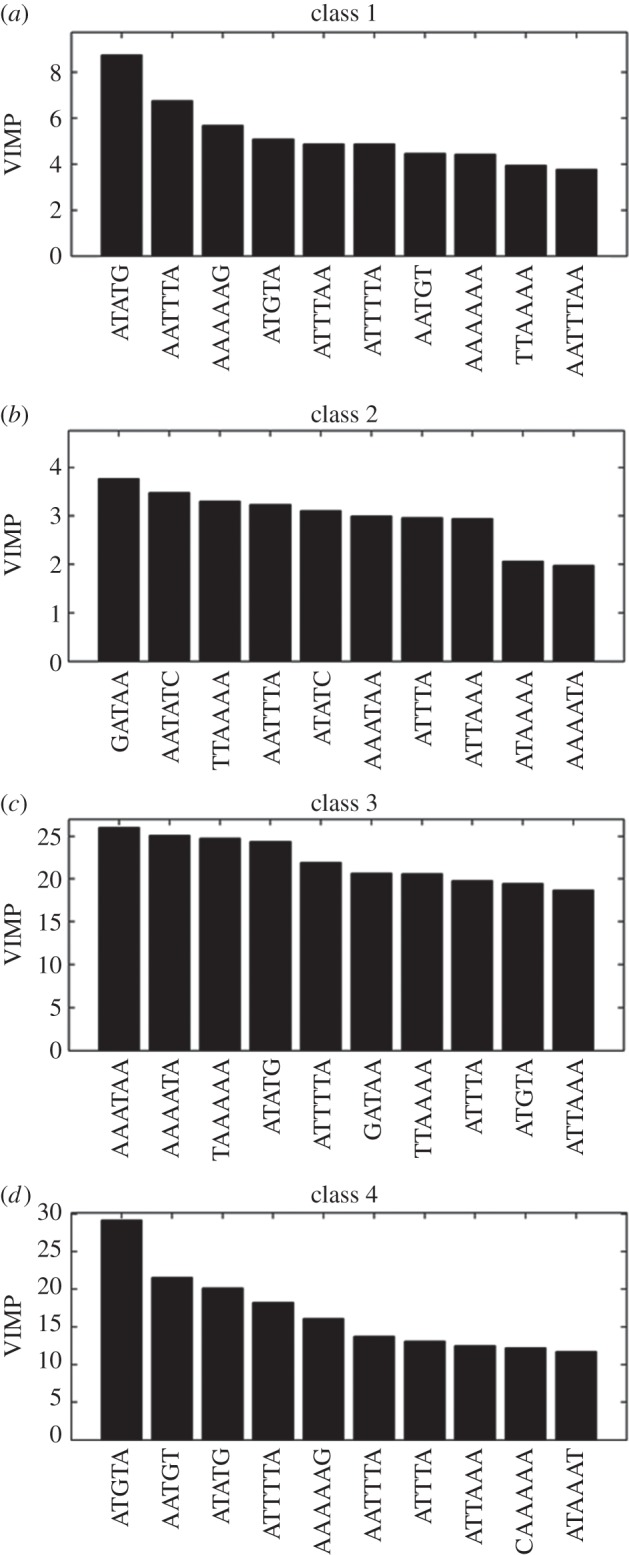
*Cis* elements defining the phase of expression for circadian genes in *Arabidopsis*. Circadian genes identified in *Arabidopsis* circadian time-course data were divided into four phase modules based on projection onto circadian ICA components. RF was used to identify *cis* elements which collectively act as classifiers for each of the four modules. Classes 1 and 2 (*a* and *b*) correspond to subjective dawn and subjective dusk, respectively (second component); classes 3 and 4 (*c* and *d*) correspond to the middle of the subjective day and night, respectively (first component). Motifs were selected and ordered through their VIMP.

We then sought to make use of this novel dataset to extend our characterization of the circadian system by looking for *cis* elements which could explain patterns of expression in the *Arabidopsis* data. For this, we also applied a novel approach. Building on recent research suggesting that multiple elements, in fact, act coordinately to generate a specific circadian pattern, we used a method which would identify such coordinately acting groups [8]. For effective utilization of the large number of *cis* elements likely to be involved in such multi-variate responses, the RF methodology was used to predict important motifs. One-kilobase-pair lengths of promoter sequence were analysed and all possible 5–8-mer sequences were considered. Of these, the 21 sequences showing the highest enrichment as isolated elements in the circadian dataset were chosen for the combinatorial analysis (electronic supplementary material, table S1). RF seeks to assign new samples to specific groups or classes based on features in common with other members of that class—in this case, *cis* elements. It uses a decision tree system of classifying, that is, it asks whether one feature at a time is present or not, thus producing two branches. These branches then branch further as additional features are considered. Ultimately, these decisions about the features of a sample lead to its assignment to a specific class. In an RF, an ensemble of decision trees is created. Each individual tree is grown from a randomly sampled subspace of input features (*cis* elements from among the 21 highest enriched individual elements) and final classification is made by combining results from trees via voting. It is a machine-learning approach which, therefore, makes use of subsets of data to capture these features of interest. The learning element of this approach comes from the way in which these decision trees are initially created using two-thirds the dataset. The decision tree is then re-created using the remaining subset of the data to assess whether the same classifiers can correctly assign the members of this subset. If not, a new decision tree is created. Figure 6 depicts the top 10 individual *cis* element motifs identified by RF, ordered by VIMP (the relative contribution of that variable or motif to the classification of the genes) for each of the four phase modules. The results here are combinatorial in that the data represent a group of *cis* elements which, in the context of other elements, are predicted to faithfully determine the phase of the genes within each phase module. Notably, several of the detected *cis* sequences giving the highest VIMP in combinatorial analysis form part of the elements previously described in the literature; for example, AATATC, part of the evening element, involved in regulation of a number of circadian genes in *Arabidopsis* [23]; GATAA, part of the I-box, involved in response to light [24]; and CAAAA, part of the CAB2 DET1-associated factor 1 binding site (CDA-1) in the dark response element, involved in response to darkness [25]. Some elements which might have been expected based on previous phase grouping analyses were not identified; notably, the G-box (CACGTG), the telo-box (AAACCCT) or the starch box (AGCCC) [26]. It should be stressed that these were not among the 21 highest enriched individual elements used for our combinatorial analysis. However, this was not unexpected as our initial overrepresentation analysis was carried out using the full set of rhythmic genes rather than the phase-specific sets used by Michael *et al.* [26].

The overall performance of RF can more objectively be assessed using ROC curves. An ROC curve shows the false-positive rate (the number of false-positive predictions, as a proportion of the total number of negative predictions) along the *x*-axis and the true-positive rate (the number of correctly predicted positive predictions, as a proportion of the total number of positive predictions) along the *y*-axis. A perfect ROC curve would be a horizontal line *y* = 1. A common metric for assessment of performance is the area under the curve (AUC) value. Electronic supplementary material, figure S2, portrays ROC curves for the ability of the identified *cis* elements to correctly predict the four phase modules. The AUC values for the four classes are 0.9420, 0.9429, 0.9567and 0.9449. A very high AUC in all cases indicates that the identified combinations of promoter elements can be considered meaningful with a high degree of confidence.

### 3.5. Progressive combinations of elements predict phase

**Figure 7.**
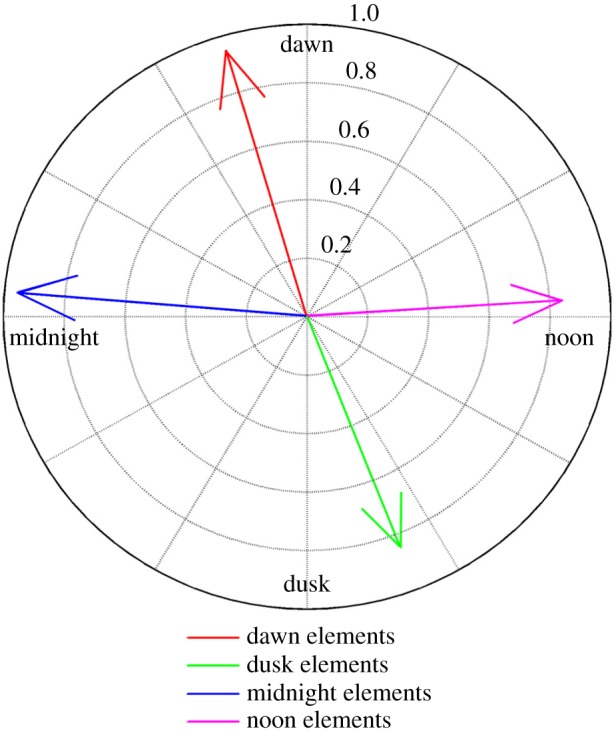
Mean phase of genes possessing progressive *cis* element combinations identified by RF. The tips of the arrows represent the mean dot product projection of each of the four groups of genes possessing the distinct *cis* element combinations identified in table 1 onto the first (*x*-axis) and second (*y*-axis) circadian ICA components.

In order to test our hypothesis that progressive combinations of individual *cis* elements acting in concert could be responsible for the range of possible phases of *Arabidopsis* circadian output genes, we compared the elements associated with each phase module. We did this by aligning the elements common to more than one phase classifier, along with the previously highlighted elements of interest, in a simple presence/absence table. We observed that individual elements making up the group associated with each phase changed progressively from one phase module to the next (table 1). Moving from one phase to the next through the day sees the gradual addition and/or removal of elements in a sequence. For example, addition of GATAA to the dawn-phased element combination, and removal of AAAAG, AATGT and AATTTA, shifts the timing of genes from the dawn to noon. In order to confirm the significance of the element groups described here, we identified the four groups of our circadian genes containing the element groups described in table 1 and analysed their mean phase. The mean position of each group of genes, when projected onto the two key circadian eigentrends defined by ICA, is represented in figure 7. For each group of genes, the mean phase is very close to the component axis representing the expected phase based on their element combinations.

**Table 1.** Comparison of *cis* elements classifying phase of expression. *Cis* elements contributing to more than one phase of expression or forming part of previously recognized light or circadian elements are shown for each of the four phase modules based on projection onto circadian ICA components. Phases are double plotted to highlight the progressive cyclic pattern of element contribution.

	AATATC	ATATC	GATAA	ATTTA(A)	ATATG	ATGTA	ATTTTA	AAAAG	AATGT	AATTTA	CAAAA
midnight				✓	✓	✓	✓	✓	✓	✓	✓
dawn				✓	✓	✓	✓	✓	✓	✓	
noon			✓	✓	✓	✓	✓				
dusk	✓	✓	✓	✓						✓	
midnight				✓	✓	✓	✓	✓	✓	✓	✓
dawn				✓	✓	✓	✓	✓	✓	✓	
noon			✓	✓	✓	✓	✓				
dusk	✓	✓	✓	✓						✓

## 4. Discussion

We set out to test the hypothesis that the full range of phases of circadian output genes in *Arabidopsis* could be realized via the progressive variation of *cis* element combinations. Previous research looking for phase-specific promoter elements has focused on the identification of overrepresented individual elements explaining a single phase of expression [23,26], but the expression patterns of a large proportion of clock output genes remains unexplained. To test our hypothesis, we took a combinatorial approach to identify potential promoter elements acting in concert to define the time of expression of phase groups of circadian genes as a whole.

### 4.1. Independent component analysis as a method of identifying circadian genes in short time-course microarray data

Identification of meaningful phase-specific groups of circadian genes is an essential prerequisite to analysis of promoter elements. Previously used algorithms have included robust periodicity testing, Bayesian mixture models, regression-based matching to model functions, Fisher's *G* testing, the Lomb–Scargle periodogram, Fourier transformation and the Laplace periodogram among others employed [9]. Conceptually, the methods encompass two primary categories: pattern matching in the time domain, or signal decomposition or filtering in the frequency domain. In the case of pattern-matching approaches, the patterns are predetermined and, therefore, biased. As such they may not truly reflect the dominant expression patterns among the oscillating genes produced by the action of the driving transcription factors. Signal decomposition methods look for frequencies of approximately 24 h in an unbiased manner but their accuracy is strongly linked to the duration of the time-course data available. For short time courses of 2 days, which are commonly used for circadian microarray experiments, these methods have limited power to identify circadian genes with certainty. These methods to identify circadian genes also suffer from the drawback that they do not naturally sort genes into phase groups; instead, phase groups for subsequent analysis are usually imposed artificially. Such approaches to the identification of circadian genes in microarray data have yielded quite different sets of genes and, as such, there is no gold standard set of circadian genes in *Arabidopsis* [9].

Here, we have identified an additional approach which is suited to our aim of sorting genes into phase groupings based upon inherent patters in the data. We identified a novel, unbiased method for the identification of genes showing circadian expression in short time-course, relatively low-resolution microarray data using a method of ICA to identify components in global gene expression. We followed this by projection of individual gene expression patterns onto these components. Significantly, ICA identified two orthogonal components which accounted for the majority of variance in the gene expression data. Projection of individual gene expression data onto these components showed that known circadian clock-regulated genes showed a circular distribution around the edge of the scatter plot, correlating well with one or both of the components. When we compared our approach with that of Covington *et al*. [16], which used the regression-based COSOPT method [18], we observed that, although we identified a similar total number of circadian genes (2948 compared with the 2885 identified by Covington *et al.*), our method identified only 1164 genes in common with the COSOPT method. We do not propose that those genes identified as circadian by other methods are incorrectly labelled. Many genes identified as circadian show relatively low amplitude, meaning that their identification is particularly sensitive to the method used. It is, however, encouraging that our method identifies all 109 distinct circadian genes identified in the same dataset when we applied the very stringent Lomb–Scargle periodogram approach [9] (electronic supplementary material, table S2). Our method naturally sorts circadian genes into phase groups according to closeness to one of the inherent orthogonal components that we identified. It is, thus, mathematically distinct from other methods and would, therefore, be expected to select differently, particularly among low-amplitude cycling genes. Our aim was to produce a method that was unbiased and, therefore, reflected patterns inherent in the data for the purpose of sorting genes.

It is tempting to speculate that the two orthogonal components identified may be indicative of underlying biological orthogonality inherent in the way that plant rhythms are generated. One can conjecture that each eigentrend represents a regulatory phase pattern that is biologically interpretable. It is important to stress that biological orthogonality does not imply distinct oscillators. Components of distinct oscillators could operate with non-orthogonal phases. Instead, we propose that the driving factors behind the module groups are orthogonal in terms of phase.

The extent of conservation of our four modules across species is striking in terms both of the patterns and of the constituents of the modules. This adds further significance to the observation. Orthologues of the vast majority of clock genes in *Arabidopsis* have been isolated in a wide range of species, including rice and maize, suggesting a common mechanism driving rhythmicity. Capturing the module–module interactions also, therefore, takes on a higher priority as it will probably allow any findings to be applied across the plant kingdom.

### 4.2. Promoter element combinations determining phase

Grouping of genes into four phase modules according to positive or negative correlation with the two components was then used for a novel combinatorial search for promoter elements responsible for the phase of expression of circadian genes in *Arabidopsis*. We used the ensemble machine-learning approach RF. This decision tree method has the advantage of being able to consider the combinatorial contribution of multiple promoter *cis* elements to the overall expression pattern of a gene. This is an important consideration in the search for circadian *cis* elements in the light of recent findings published elsewhere as to the nature of transcriptional control of the pattern of circadian gene expression in *Arabidopsis* [8]. The elements identified as most important in combination with others included a number of entirely novel elements but also some that are part of elements previously identified as conferring phase information in isolation, such as the evening element.

We showed that, for each module, combinations of these promoter features could be identified as being able to provide sufficient information to allow genes to be assigned to the correct module with high precision. Projection of the four groups of genes containing these element combinations onto the two circadian eigentrends identified by ICA demonstrated that these four groups did, indeed, show the mean phase predicted by these element combinations. In further support of the merit of our results, the previously highlighted elements of interest occur in phase module groups consistent with the known function of the elements of which they form a part. AATATC, part of the evening element, occurred in the ‘dusk’ (class 2) phase group; GATAA, part of the I-box, occurred in the ‘noon’ (class 3) and ‘dusk’ (class 2) phase groups; and CAAAA, part of the CDA-1, occurred in the ‘midnight’ (class 4) phase group. Sequences of 5–8 base pairs were chosen for the analysis as this range provided the best results. Although some previously identified circadian elements such as the evening element [23] are longer in full, we found that increasing the length of the target sequence did not further increase the precision.

### 4.3. Progressive interactions between promoter elements associate with sequential phases

A number of elements were common to more than one phase module group and we observed that these element groupings changed in a progressive pattern from one phase group to the next. Removal of certain elements or addition of others was found to change the predicted phase of a gene in a consistent manner. This finding suggests that the phase of a gene is determined by the additive effect of multiple transcription factors peaking at specific points throughout the circadian day. Our hypothesis ordains that a gene upregulated by a dawn-phased transcription factor would begin to be transcribed just as that transcription factor accumulates. Although the influence of that transcription factor will begin to wane after dawn, if the target gene is also upregulated by a noon-phased transcription factor it would see a simultaneous increase in the influence of this noon-phased transcription factor which would begin to accumulate just following dawn. Assuming both transcription factors show a sigmoidal pattern of fluctuation, their net maximum positive effect will occur at the midpoint between the peaks of the two. Thus the target gene will possess a phase intermediate between the two. Such a system would allow a relatively small number of drivers to achieve a large distribution of phases. Equally, a precisely graduated range of phases among target genes could be determined by the accumulated weight of transcription factor influences. If a gene were upregulated by two dawn-phased transcription factors and one noon-phased transcription factor its net phase would be expected to be nearer to dawn than to noon. Our analysis is based upon division of the circadian gene set into four phase groupings. It is likely that the true situation, a continuum of phases, is achieved by a gradual sliding scale of presence of these progressive combinations of transcription factor binding sites.

## Supplementary Material

Smieszek et al Supplementary data
