# Supplementary material for: Progressive promoter element combinations classify conserved orthogonal plant circadian gene expression modules
Source: J R Soc Interface. 2014 Oct 6;11(99):20140535. doi: 10.1098/rsif.2014.0535 (PMC4233729; doi:10.1098/rsif.2014.0535)
Supplement: Smieszek et al Supplementary data [file rsif20140535supp1.pdf]

Progressive promoter element combinations classify conserved orthogonal plant circadian gene expression modules

Sandra P Smieszek, Haixuan Yang, Alberto Paccanaro, Paul F Devlin

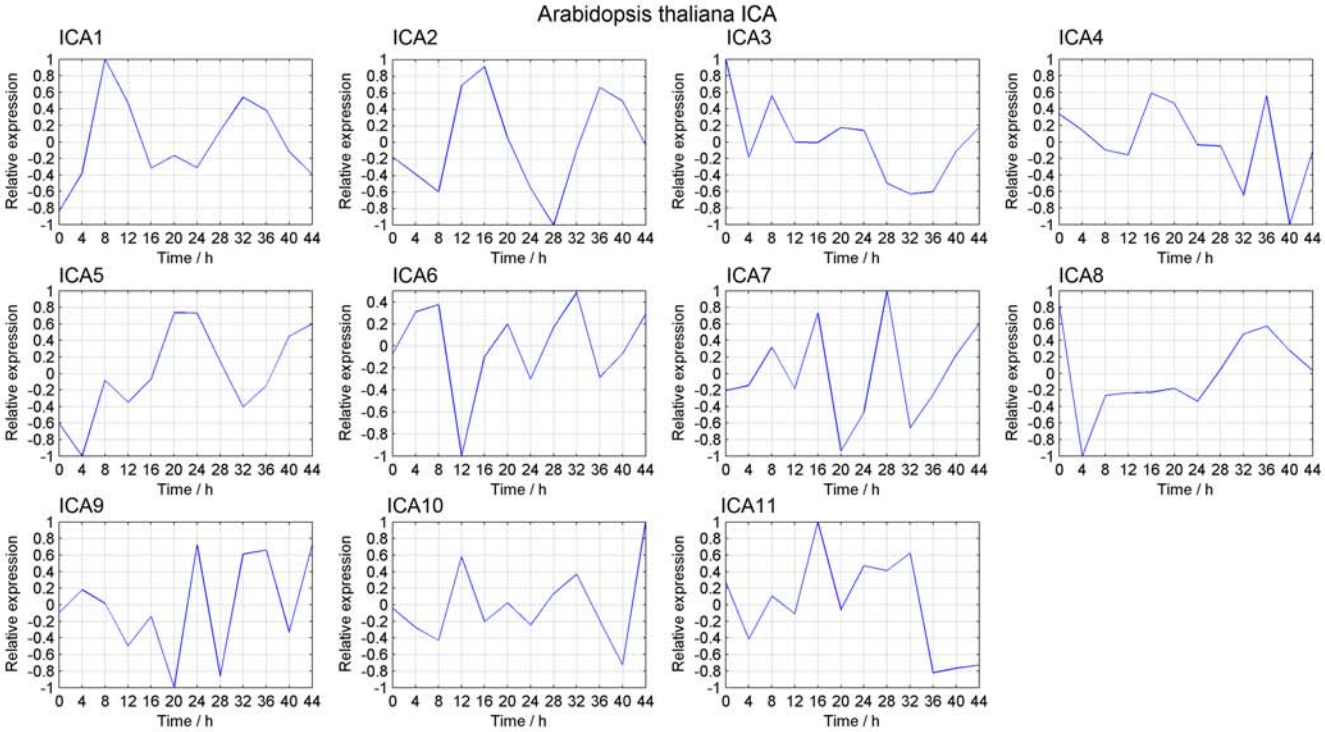

Fig. S1. ICA performed on Arabidopsis circadian microarray time series. All independent components ordered by kurtosis.

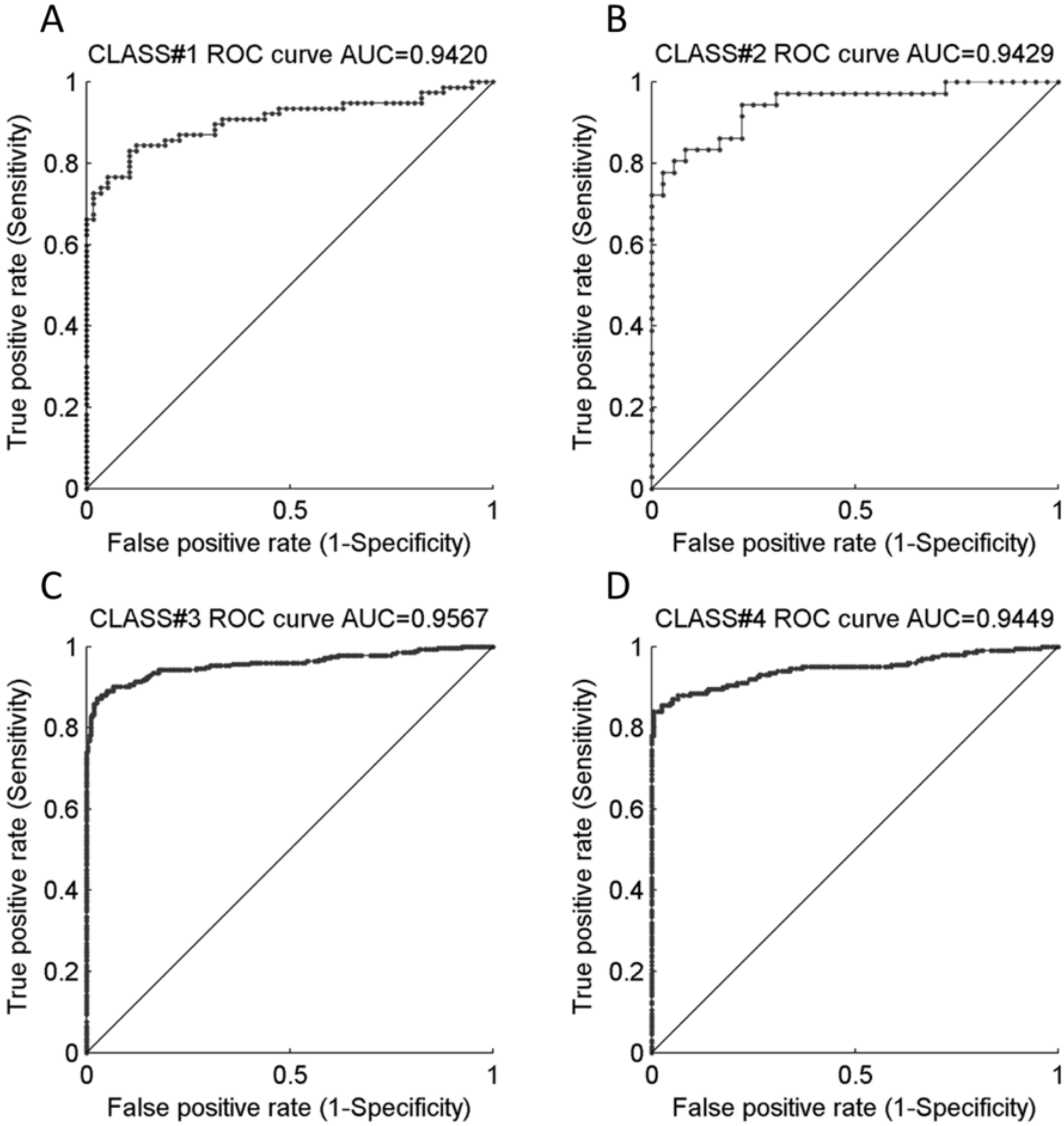

Fig. S2. Assessment of cis element classification performance. Receiver operating characteristic (ROC) curves representing the power of the feature combination classifiers identified by Random Forest to correctly assign genes to four phase modules. Classes 1 and 2 (A and B) correspond to subjective dawn and subjective dusk-phased genes respectively, whereas classes 3 and 4 (C and D) correspond to the middle of the subjective day and night respectively. The area under the curve (AUC) scores are also represented

Table S2. Genes identified as circadian. Circadian genes were selected by projection onto the two circadian eigentrends identified by ICA performed on Arabidopsis circadian microarray time series. A radial cut-off at a distance of 0.8 was applied to define the circadian gene set. Presence "1" or absence "0" for each gene is shown according to presence in the list of circdian genes selected by Covington et al., 2008, Genome Biol 9, R130 using a regression-based method; or in the list of circadian genes identified via a Lomb Scargle periodogram-based method.

Unique Gene IDs Smieszek	Covington et al	Lomb Scargle
AT1G01050	0	0
AT1G01060	1	1
AT1G01240	1	0
AT1G01440	0	0
AT1G01490	1	0
AT1G01520	1	0
AT1G01620	1	0
AT1G01770	0	0
AT1G01960	0	0
AT1G01970	1	0
AT1G02000	0	0
AT1G02120	0	0
AT1G02270	0	0
AT1G02300	1	0
AT1G02340	1	0
AT1G02640	1	0
AT1G02660	0	0
AT1G02820	1	0
AT1G02860	0	0
AT1G03055	0	1
AT1G03090	1	0
AT1G03290	0	0
AT1G03520	0	0
AT1G03590	0	0
AT1G03610	0	0
AT1G03680	1	0
AT1G03740	0	0
AT1G03820	0	0
AT1G03850	0	0
AT1G03910	0	0
AT1G03960	1	0
AT1G04130	0	0
AT1G04170	1	0
AT1G04220	0	0
AT1G04290	0	0

AT1G04300	0	0
AT1G04400	1	0
AT1G04440	0	0
AT1G04480	0	0
AT1G04520	0	0
AT1G04530	1	1
AT1G04550	0	0
AT1G04690	0	0
AT1G04710	1	0
AT1G04780	0	0
AT1G04870	1	0
AT1G04950	0	0
AT1G05000	0	0
AT1G05030	0	0
AT1G05170	1	0
AT1G05200	0	0
AT1G05360	0	0
AT1G05380	0	0
AT1G05385	0	0
AT1G05570	0	0
AT1G05690	0	0
AT1G05710	0	0
AT1G05805	1	0
AT1G05810	0	0
AT1G05850	0	0
AT1G05900	0	0
AT1G05960	0	0
AT1G06000	1	0
AT1G06160	0	0
AT1G06410	0	0
AT1G06430	0	0
AT1G06450	0	0
AT1G06460	1	0
AT1G06550	1	0
AT1G06570	1	0
AT1G06690	1	0
AT1G06830	0	0
AT1G06960	0	0
AT1G07010	1	1
AT1G07030	0	0
AT1G07040	1	0
AT1G07050	1	1
AT1G07070	0	0
AT1G07180	1	0
AT1G07280	1	0
AT1G07420	1	0
AT1G07570	0	0
AT1G07650	0	0
AT1G07700	1	0
AT1G07830	0	0

AT1G08220	0	0
AT1G08250	1	0
AT1G08360	0	0
AT1G08380	1	0
AT1G08450	0	0
AT1G08550	1	0
AT1G08660	0	0
AT1G08680	0	0
AT1G08720	0	0
AT1G08980	1	0
AT1G09200	0	0
AT1G09250	0	0
AT1G09280	0	0
AT1G09310	1	0
AT1G09320	1	0
AT1G09420	0	0
AT1G09430	0	0
AT1G09460	0	0
AT1G09570	1	0
AT1G09575	0	0
AT1G09680	0	0
AT1G09750	1	0
AT1G09780	0	0
AT1G09840	0	0
AT1G10020	1	0
AT1G10060	0	0
AT1G10070	0	0
AT1G10090	1	0
AT1G10140	1	0
AT1G10200	1	0
AT1G10310	0	0
AT1G10370	1	0
AT1G10522	1	0
AT1G10610	0	0
AT1G10650	1	0
AT1G10740	0	0
AT1G10760	1	0
AT1G10900	1	0
AT1G10960	1	0
AT1G10990	0	0
AT1G11210	1	0
AT1G11260	1	0
AT1G11330	0	0
AT1G11360	1	0
AT1G11380	0	0
AT1G11545	0	0
AT1G11630	0	0
AT1G11700	0	0
AT1G11790	0	0
AT1G11840	1	0

AT1G11850	0	0
AT1G12020	0	0
AT1G12120	1	0
AT1G12230	1	0
AT1G12370	0	0
AT1G12500	1	0
AT1G12580	1	0
AT1G12680	0	0
AT1G12710	1	0
AT1G12730	0	0
AT1G12780	1	0
AT1G12845	1	0
AT1G12880	1	0
AT1G13080	1	0
AT1G13210	0	0
AT1G13270	1	0
AT1G13440	1	0
AT1G13560	0	0
AT1G13570	0	0
AT1G13650	1	0
AT1G13940	0	0
AT1G13990	1	0
AT1G14150	1	0
AT1G14280	1	0
AT1G14290	1	0
AT1G14320	0	0
AT1G14330	0	0
AT1G14350	0	0
AT1G14360	0	0
AT1G14380	0	0
AT1G14620	0	0
AT1G14685	1	0
AT1G14740	0	0
AT1G14920	1	0
AT1G14980	0	0
AT1G14990	0	0
AT1G15060	0	0
AT1G15100	1	0
AT1G15220	0	0
AT1G15290	0	0
AT1G15340	0	0
AT1G15350	1	0
AT1G15380	1	0
AT1G15410	0	0
AT1G15670	0	0
AT1G15710	0	0
AT1G15780	0	0
AT1G15800	0	0
AT1G15930	1	0
AT1G15950	1	0

AT1G16060	0	0
AT1G16080	1	0
AT1G16180	1	0
AT1G16310	0	0
AT1G16390	0	0
AT1G16720	1	0
AT1G16730	0	0
AT1G16840	0	0
AT1G16880	1	0
AT1G17050	1	1
AT1G17090	0	0
AT1G17100	1	0
AT1G17145	0	0
AT1G17170	0	0
AT1G17360	1	0
AT1G17460	1	0
AT1G17500	0	0
AT1G17560	0	0
AT1G17665	0	0
AT1G17745	0	0
AT1G17880	0	0
AT1G17970	0	0
AT1G17980	0	0
AT1G18060	1	0
AT1G18210	0	0
AT1G18330	1	0
AT1G18460	0	0
AT1G18470	1	0
AT1G18480	0	0
AT1G18500	0	0
AT1G18570	1	0
AT1G18590	0	0
AT1G18600	0	0
AT1G18620	1	0
AT1G18720	1	0
AT1G18740	1	0
AT1G18810	1	0
AT1G19050	0	0
AT1G19140	0	0
AT1G19310	0	0
AT1G19370	1	0
AT1G19450	1	0
AT1G19540	0	0
AT1G19700	0	1
AT1G19710	0	0
AT1G19715	1	0
AT1G19740	0	0
AT1G19835	0	0
AT1G19920	1	0
AT1G19970	0	0

AT1G20010	1	0
AT1G20020	1	0
AT1G20070	1	0
AT1G20390	0	0
AT1G20440	1	0
AT1G20620	1	0
AT1G20630	1	0
AT1G20650	1	0
AT1G20823	0	0
AT1G20830	0	0
AT1G20840	0	0
AT1G20880	0	0
AT1G21000	0	0
AT1G21060	1	0
AT1G21130	1	0
AT1G21250	1	0
AT1G21410	0	0
AT1G21460	1	0
AT1G21500	1	0
AT1G21560	0	0
AT1G21610	0	0
AT1G21640	0	0
AT1G21670	1	0
AT1G21680	1	0
AT1G21730	0	0
AT1G21830	0	0
AT1G21920	0	0
AT1G22060	0	0
AT1G22070	1	0
AT1G22160	0	0
AT1G22330	0	0
AT1G22360	0	0
AT1G22370	1	0
AT1G22410	1	0
AT1G22530	0	0
AT1G22540	0	0
AT1G22570	1	0
AT1G22590	0	0
AT1G22740	0	0
AT1G22770	1	1
AT1G22790	0	0
AT1G22850	1	0
AT1G22870	0	0
AT1G22890	1	0
AT1G22910	0	0
AT1G23030	0	0
AT1G23060	1	0
AT1G23080	1	0
AT1G23190	0	0
AT1G23205	1	0

AT1G23220	0	0
AT1G23330	0	0
AT1G23390	1	0
AT1G23410	0	0
AT1G23550	0	0
AT1G23740	1	0
AT1G23750	0	0
AT1G23870	1	0
AT1G24020	1	0
AT1G24070	0	0
AT1G24100	1	0
AT1G24180	0	0
AT1G24190	0	0
AT1G24280	0	0
AT1G24290	0	0
AT1G24360	0	0
AT1G24440	1	0
AT1G24625	0	0
AT1G25520	0	1
AT1G25540	0	0
AT1G26100	0	0
AT1G26150	0	0
AT1G26210	0	0
AT1G26220	0	0
AT1G26230	1	1
AT1G26440	0	0
AT1G26560	1	0
AT1G26580	0	0
AT1G26620	0	0
AT1G26770	0	0
AT1G26820	0	0
AT1G26880	0	0
AT1G26920	1	0
AT1G27090	0	0
AT1G27120	1	0
AT1G27340	0	0
AT1G27385	0	0
AT1G27400	1	0
AT1G27450	1	0
AT1G27480	1	0
AT1G27520	0	0
AT1G27530	0	0
AT1G27600	0	0
AT1G27630	1	0
AT1G27650	1	0
AT1G27760	0	0
AT1G27950	0	0
AT1G27970	0	0
AT1G28010	0	0
AT1G28050	1	0

AT1G28070	0	0
AT1G28200	0	0
AT1G28260	0	0
AT1G28320	0	0
AT1G28330	1	0
AT1G28395	1	0
AT1G28410	0	0
AT1G28510	0	0
AT1G28560	0	0
AT1G28570	0	1
AT1G28600	1	1
AT1G28610	0	0
AT1G28680	0	0
AT1G29120	0	0
AT1G29250	0	0
AT1G29530	0	0
AT1G29700	1	1
AT1G30070	0	0
AT1G30110	0	0
AT1G30200	0	0
AT1G30270	0	0
AT1G30360	0	0
AT1G30530	1	0
AT1G30620	0	0
AT1G30820	0	0
AT1G31130	1	0
AT1G31160	0	0
AT1G31190	1	0
AT1G31300	0	0
AT1G31480	0	0
AT1G31550	0	0
AT1G31580	1	0
AT1G31690	1	0
AT1G31850	1	0
AT1G31860	1	0
AT1G31920	0	0
AT1G32090	0	0
AT1G32160	0	0
AT1G32170	0	0
AT1G32220	1	0
AT1G32260	0	0
AT1G32340	0	0
AT1G32470	1	0
AT1G32520	1	0
AT1G32550	1	0
AT1G32700	0	0
AT1G32780	0	0
AT1G32790	1	0
AT1G32870	1	0
AT1G32900	1	1

AT1G32920	0	0
AT1G33050	0	0
AT1G33110	1	0
AT1G33240	1	0
AT1G33260	1	0
AT1G33490	0	0
AT1G33560	0	0
AT1G33590	1	0
AT1G33810	0	0
AT1G33970	1	0
AT1G33980	0	0
AT1G34000	1	0
AT1G34030	0	0
AT1G34150	0	0
AT1G34300	1	0
AT1G34310	1	0
AT1G34340	1	0
AT1G34370	0	0
AT1G34380	0	0
AT1G34430	0	0
AT1G34630	0	0
AT1G34760	0	0
AT1G35260	0	0
AT1G35290	0	0
AT1G35560	1	1
AT1G35580	0	0
AT1G35620	0	0
AT1G36050	0	0
AT1G36370	0	0
AT1G36940	0	0
AT1G41830	1	0
AT1G42470	0	0
AT1G42540	0	0
AT1G43560	0	0
AT1G43620	1	0
AT1G43790	1	0
AT1G44000	1	0
AT1G44100	0	0
AT1G44350	1	0
AT1G44446	1	1
AT1G44575	1	0
AT1G44810	0	0
AT1G45150	1	0
AT1G45474	0	0
AT1G45688	1	0
AT1G45976	0	0
AT1G46264	0	0
AT1G47250	0	0
AT1G47260	0	0
AT1G47270	0	0

AT1G47400	0	0
AT1G47530	1	0
AT1G47570	0	0
AT1G47710	0	0
AT1G48040	0	0
AT1G48100	0	0
AT1G48170	0	0
AT1G48200	0	0
AT1G48210	1	0
AT1G48260	1	0
AT1G48320	0	0
AT1G48330	1	0
AT1G48780	0	0
AT1G48840	0	0
AT1G48960	0	0
AT1G49200	1	0
AT1G49410	0	0
AT1G49430	0	0
AT1G49660	0	0
AT1G49720	1	0
AT1G49730	0	0
AT1G50020	1	0
AT1G50030	0	0
AT1G50320	1	0
AT1G50410	0	0
AT1G50575	1	0
AT1G51060	0	0
AT1G51070	1	0
AT1G51140	1	0
AT1G51570	0	0
AT1G51610	1	0
AT1G51680	1	0
AT1G51700	1	0
AT1G51730	0	0
AT1G51940	1	0
AT1G52050	0	0
AT1G52190	0	0
AT1G52290	0	0
AT1G52510	0	0
AT1G52590	1	0
AT1G52600	0	0
AT1G52670	0	0
AT1G52720	1	0
AT1G52870	0	0
AT1G53000	0	0
AT1G53035	0	0
AT1G53060	0	0
AT1G53070	0	0
AT1G53090	1	1
AT1G53120	0	0

AT1G53165	0	0
AT1G53320	1	0
AT1G53390	0	0
AT1G53430	1	0
AT1G53440	0	0
AT1G53580	1	0
AT1G53590	1	0
AT1G53910	0	0
AT1G54060	0	0
AT1G54100	0	0
AT1G54130	1	0
AT1G54200	0	0
AT1G54690	0	0
AT1G54740	1	0
AT1G54780	1	0
AT1G54790	0	0
AT1G54830	0	0
AT1G55210	1	0
AT1G55250	0	0
AT1G55260	1	0
AT1G55265	0	0
AT1G55310	0	0
AT1G55510	0	0
AT1G55590	0	0
AT1G55670	1	0
AT1G55890	1	0
AT1G55910	1	1
AT1G55960	1	1
AT1G56170	1	0
AT1G56220	1	0
AT1G56300	1	0
AT1G56430	0	0
AT1G56500	0	0
AT1G56510	0	0
AT1G56580	1	0
AT1G56670	0	0
AT1G56720	1	0
AT1G57680	1	0
AT1G57770	0	0
AT1G58110	1	0
AT1G58170	1	0
AT1G58200	0	0
AT1G58290	1	0
AT1G59610	0	0
AT1G59700	1	0
AT1G59840	0	0
AT1G59870	1	0
AT1G59970	0	0
AT1G60140	1	0
AT1G60160	0	0

AT1G60270	1	0
AT1G60390	0	0
AT1G60860	1	0
AT1G60940	0	0
AT1G60950	0	0
AT1G61040	0	0
AT1G61065	0	0
AT1G61100	0	0
AT1G61260	0	0
AT1G61690	0	0
AT1G61890	1	0
AT1G61900	0	0
AT1G62180	1	0
AT1G62250	1	0
AT1G62380	0	0
AT1G62430	1	0
AT1G62480	0	0
AT1G62540	1	0
AT1G62560	0	0
AT1G62750	1	0
AT1G62780	1	0
AT1G62960	1	0
AT1G63000	0	0
AT1G63110	0	0
AT1G63120	0	0
AT1G63240	0	0
AT1G63610	1	0
AT1G63690	0	0
AT1G63780	1	0
AT1G64040	0	0
AT1G64170	0	0
AT1G64230	0	0
AT1G64400	1	0
AT1G64460	0	0
AT1G64490	0	0
AT1G64500	1	1
AT1G64530	0	0
AT1G64670	0	0
AT1G64740	0	0
AT1G64770	1	0
AT1G64780	1	0
AT1G64860	1	0
AT1G64890	0	0
AT1G64900	1	0
AT1G64970	0	0
AT1G64980	0	0
AT1G65060	1	0
AT1G65220	0	0
AT1G65280	0	0
AT1G65290	0	0

AT1G65490	1	0
AT1G65560	1	0
AT1G65580	1	0
AT1G65660	0	0
AT1G65840	0	0
AT1G65860	0	0
AT1G65870	0	0
AT1G65900	1	0
AT1G65980	0	0
AT1G66080	0	0
AT1G66130	0	0
AT1G66150	1	0
AT1G66180	1	0
AT1G66230	0	0
AT1G66330	1	0
AT1G66580	1	0
AT1G66670	0	0
AT1G66820	1	0
AT1G66880	0	0
AT1G66940	0	0
AT1G67030	0	0
AT1G67060	0	0
AT1G67310	1	0
AT1G67360	1	0
AT1G67470	0	1
AT1G67480	0	0
AT1G67580	0	0
AT1G67590	0	0
AT1G67660	1	0
AT1G67730	0	0
AT1G67890	0	0
AT1G67970	1	0
AT1G68020	0	0
AT1G68080	0	0
AT1G68190	1	0
AT1G68220	0	0
AT1G68440	1	0
AT1G68470	1	0
AT1G68500	0	0
AT1G68540	0	0
AT1G68560	0	0
AT1G68570	1	0
AT1G68585	0	0
AT1G68590	0	0
AT1G68600	0	0
AT1G68660	1	0
AT1G68670	0	0
AT1G68830	1	0
AT1G68840	1	0
AT1G68920	0	0

AT1G69040	0	0
AT1G69160	1	0
AT1G69220	0	0
AT1G69295	1	0
AT1G69340	1	0
AT1G69523	1	0
AT1G69530	1	0
AT1G69570	0	1
AT1G69620	0	0
AT1G69700	0	0
AT1G69730	0	1
AT1G69740	1	0
AT1G69780	0	0
AT1G69830	1	0
AT1G70000	1	1
AT1G70230	1	0
AT1G70250	0	0
AT1G70290	1	0
AT1G70310	0	0
AT1G70420	1	0
AT1G70530	1	0
AT1G70580	1	0
AT1G70610	1	1
AT1G70620	0	0
AT1G70700	1	0
AT1G70730	0	0
AT1G70760	0	0
AT1G70820	1	0
AT1G70940	1	0
AT1G70985	0	0
AT1G71020	0	0
AT1G71030	1	0
AT1G71480	1	0
AT1G71500	1	0
AT1G71780	0	0
AT1G71810	0	0
AT1G71860	0	0
AT1G71970	1	0
AT1G71980	0	0
AT1G72030	1	0
AT1G72090	0	0
AT1G72150	1	0
AT1G72160	1	0
AT1G72180	0	0
AT1G72230	0	0
AT1G72300	0	0
AT1G72410	0	0
AT1G72420	0	0
AT1G72710	0	0
AT1G72740	0	0

AT1G72770	1	1
AT1G72790	0	0
AT1G72810	0	0
AT1G72820	0	0
AT1G73177	0	0
AT1G73200	0	0
AT1G73380	0	0
AT1G73390	0	0
AT1G73480	1	0
AT1G73630	0	0
AT1G73650	1	0
AT1G73655	1	0
AT1G73660	1	0
AT1G73670	1	0
AT1G73710	0	0
AT1G73740	1	0
AT1G73750	0	0
AT1G73760	1	0
AT1G73830	1	0
AT1G73870	1	1
AT1G73920	0	0
AT1G73980	0	0
AT1G73990	1	0
AT1G74020	0	0
AT1G74100	0	0
AT1G74230	0	0
AT1G74270	0	0
AT1G74300	0	0
AT1G74410	0	0
AT1G74680	1	0
AT1G74710	1	0
AT1G74730	0	0
AT1G74940	0	0
AT1G75090	1	0
AT1G75100	1	0
AT1G75130	1	0
AT1G75140	1	0
AT1G75180	1	0
AT1G75190	1	0
AT1G75220	0	0
AT1G75240	0	0
AT1G75270	0	0
AT1G75280	1	0
AT1G75370	1	0
AT1G75500	0	0
AT1G75540	0	0
AT1G75660	0	0
AT1G75680	0	0
AT1G75690	0	0
AT1G75710	1	0

AT1G75800	1	0
AT1G75960	0	0
AT1G76240	1	0
AT1G76320	0	0
AT1G76410	0	0
AT1G76450	0	0
AT1G76490	0	0
AT1G76570	1	0
AT1G76590	1	0
AT1G76660	0	0
AT1G76790	0	0
AT1G76990	1	0
AT1G77000	1	0
AT1G77080	0	0
AT1G77090	1	0
AT1G77210	1	0
AT1G77450	1	0
AT1G77550	0	0
AT1G77750	0	0
AT1G77870	0	0
AT1G77930	1	0
AT1G77940	0	0
AT1G78020	1	0
AT1G78080	0	0
AT1G78100	1	0
AT1G78110	0	0
AT1G78240	0	0
AT1G78270	0	0
AT1G78290	1	0
AT1G78310	0	0
AT1G78380	0	0
AT1G78460	1	0
AT1G78510	1	1
AT1G78570	0	0
AT1G78600	1	0
AT1G78700	0	0
AT1G78895	0	0
AT1G79230	0	0
AT1G79260	0	0
AT1G79270	1	0
AT1G79390	0	0
AT1G79410	0	0
AT1G79440	1	0
AT1G79460	0	0
AT1G79510	1	0
AT1G79520	1	0
AT1G79600	1	0
AT1G79670	0	0
AT1G79700	0	0
AT1G79830	0	0

AT1G80130	1	0
AT1G80230	0	0
AT1G80420	0	0
AT1G80440	1	0
AT1G80500	0	0
AT1G80510	0	0
AT1G80530	1	0
AT1G80600	0	0
AT1G80680	0	0
AT1G80760	0	0
AT1G80850	0	0
AT1G80920	1	0
AT2G01100	0	0
AT2G01170	1	0
AT2G01190	0	0
AT2G01260	1	0
AT2G01400	0	0
AT2G01540	0	0
AT2G01590	0	0
AT2G01600	0	0
AT2G01710	0	0
AT2G01720	0	0
AT2G01830	0	0
AT2G01850	0	0
AT2G01930	0	0
AT2G01940	0	0
AT2G02100	1	0
AT2G02130	0	0
AT2G02170	0	0
AT2G02220	0	0
AT2G02360	0	0
AT2G02570	0	0
AT2G02710	1	0
AT2G02740	0	0
AT2G02760	1	0
AT2G02800	1	0
AT2G03350	1	0
AT2G03510	0	0
AT2G03730	1	0
AT2G04039	1	1
AT2G04400	0	0
AT2G04570	0	0
AT2G04780	0	0
AT2G04795	0	0
AT2G04850	0	0
AT2G04940	0	0
AT2G05170	0	0
AT2G05260	0	0
AT2G05620	1	1
AT2G05630	0	0

AT2G06050	1	0
AT2G06850	0	0
AT2G06925	1	1
AT2G07050	1	0
AT2G11260	0	0
AT2G13100	0	0
AT2G13650	0	0
AT2G13690	0	0
AT2G14080	1	0
AT2G14170	0	0
AT2G14460	0	0
AT2G14660	0	0
AT2G14750	1	0
AT2G14880	1	0
AT2G15020	1	1
AT2G15080	0	0
AT2G15090	1	0
AT2G15320	0	0
AT2G15570	0	0
AT2G15760	0	0
AT2G15880	0	0
AT2G15890	1	0
AT2G15900	0	0
AT2G16070	1	0
AT2G16250	0	0
AT2G16430	0	0
AT2G16500	0	0
AT2G16600	1	0
AT2G16780	1	0
AT2G16990	0	0
AT2G17110	0	0
AT2G17220	1	0
AT2G17265	0	0
AT2G17340	0	0
AT2G17360	0	0
AT2G17440	0	0
AT2G17450	1	0
AT2G17530	0	0
AT2G17550	0	0
AT2G17760	0	0
AT2G17820	0	0
AT2G17880	0	0
AT2G18115	0	0
AT2G18170	1	0
AT2G18230	1	0
AT2G18260	0	0
AT2G18280	1	0
AT2G18290	1	0
AT2G18300	1	0
AT2G18350	0	0

AT2G18390	0	0
AT2G18670	1	0
AT2G18700	1	0
AT2G18740	0	0
AT2G19180	0	0
AT2G19450	1	0
AT2G19580	0	0
AT2G19650	1	1
AT2G19680	0	0
AT2G19750	1	0
AT2G19800	0	0
AT2G19810	0	0
AT2G19860	1	0
AT2G19940	0	0
AT2G20060	0	0
AT2G20180	0	0
AT2G20240	0	0
AT2G20450	0	0
AT2G20610	1	0
AT2G20670	1	0
AT2G20690	1	0
AT2G20750	1	0
AT2G20820	0	0
AT2G20960	0	0
AT2G21070	0	0
AT2G21130	1	0
AT2G21220	0	0
AT2G21320	1	1
AT2G21330	1	0
AT2G21340	1	0
AT2G21380	1	0
AT2G21430	1	0
AT2G21530	0	0
AT2G21600	1	0
AT2G21630	0	0
AT2G21660	1	0
AT2G21960	0	0
AT2G21970	1	0
AT2G22240	1	1
AT2G22250	1	0
AT2G22300	0	0
AT2G22360	1	0
AT2G22420	1	0
AT2G22430	1	0
AT2G22450	1	0
AT2G22540	1	0
AT2G22660	0	0
AT2G22780	0	0
AT2G22830	1	0
AT2G22980	1	0

AT2G23030	0	0
AT2G23140	0	0
AT2G23290	0	0
AT2G23320	0	0
AT2G23340	0	0
AT2G23420	1	1
AT2G23610	0	0
AT2G23670	1	0
AT2G23840	1	0
AT2G23890	0	0
AT2G23910	1	0
AT2G23930	0	0
AT2G24060	1	0
AT2G24090	0	0
AT2G24160	0	0
AT2G24190	0	0
AT2G24200	0	0
AT2G24270	1	0
AT2G24420	0	0
AT2G24540	1	1
AT2G24550	1	0
AT2G24580	0	0
AT2G24820	0	0
AT2G25000	0	0
AT2G25010	0	0
AT2G25070	1	0
AT2G25200	0	0
AT2G25210	0	0
AT2G25355	0	0
AT2G25510	1	0
AT2G25530	1	0
AT2G25760	0	0
AT2G25800	0	0
AT2G25830	1	0
AT2G25900	1	0
AT2G26000	0	0
AT2G26080	1	0
AT2G26110	0	0
AT2G26170	1	1
AT2G26180	0	0
AT2G26190	0	0
AT2G26200	1	0
AT2G26300	0	0
AT2G26350	0	0
AT2G26430	0	0
AT2G26440	0	0
AT2G26510	0	0
AT2G26530	1	0
AT2G26640	0	0
AT2G26690	0	0

AT2G26710	1	0
AT2G26800	1	0
AT2G26900	1	0
AT2G26910	1	0
AT2G26920	0	0
AT2G27050	1	0
AT2G27060	0	0
AT2G27200	0	0
AT2G27290	0	0
AT2G27360	1	0
AT2G27420	0	1
AT2G27500	0	0
AT2G27510	0	0
AT2G27530	0	0
AT2G27550	1	0
AT2G27730	0	0
AT2G27820	0	0
AT2G27970	0	0
AT2G28080	0	0
AT2G28120	0	0
AT2G28200	1	0
AT2G28305	0	0
AT2G28320	0	0
AT2G28330	0	0
AT2G28360	0	0
AT2G28370	0	0
AT2G28450	0	0
AT2G28510	0	0
AT2G28800	0	0
AT2G28840	1	0
AT2G28900	1	0
AT2G29180	0	0
AT2G29450	0	0
AT2G29490	0	0
AT2G29530	0	0
AT2G29570	0	0
AT2G29630	1	0
AT2G29650	1	0
AT2G29670	1	0
AT2G30010	0	0
AT2G30040	0	0
AT2G30140	0	0
AT2G30160	0	0
AT2G30490	1	0
AT2G30520	1	0
AT2G30600	0	0
AT2G30695	0	0
AT2G30860	0	0
AT2G30930	0	0
AT2G30990	0	0

AT2G31040	1	0
AT2G31070	1	0
AT2G31090	0	0
AT2G31110	0	0
AT2G31150	0	0
AT2G31380	1	1
AT2G31450	0	0
AT2G31510	0	0
AT2G31670	1	0
AT2G31750	1	0
AT2G31800	1	0
AT2G31810	0	0
AT2G31880	0	0
AT2G32060	1	0
AT2G32100	1	1
AT2G32220	0	0
AT2G32380	1	0
AT2G32450	1	0
AT2G32560	0	0
AT2G32640	0	0
AT2G32710	0	0
AT2G32765	0	0
AT2G32800	0	0
AT2G32990	0	0
AT2G33150	0	0
AT2G33180	0	0
AT2G33250	1	0
AT2G33380	1	0
AT2G33430	1	0
AT2G33530	1	0
AT2G33540	0	0
AT2G33590	1	0
AT2G33700	0	0
AT2G33810	0	0
AT2G33830	1	0
AT2G34050	0	0
AT2G34090	0	0
AT2G34200	0	0
AT2G34310	1	0
AT2G34460	1	0
AT2G34480	0	0
AT2G34490	1	0
AT2G34630	1	0
AT2G34660	1	0
AT2G34690	1	0
AT2G34720	0	0
AT2G35050	0	0
AT2G35060	1	0
AT2G35230	0	0
AT2G35260	1	0

AT2G35470	0	0
AT2G35605	0	0
AT2G35650	0	0
AT2G35680	0	0
AT2G35800	0	0
AT2G35860	0	0
AT2G35880	0	0
AT2G35960	0	0
AT2G36050	0	0
AT2G36170	0	0
AT2G36230	0	0
AT2G36290	1	0
AT2G36320	1	0
AT2G36390	1	0
AT2G36530	0	0
AT2G36630	1	0
AT2G36835	1	0
AT2G36870	1	0
AT2G36910	0	0
AT2G37040	1	0
AT2G37190	1	0
AT2G37240	1	0
AT2G37250	0	0
AT2G37520	0	0
AT2G37570	0	0
AT2G37680	1	0
AT2G37760	0	0
AT2G37770	0	0
AT2G37790	1	0
AT2G37840	0	0
AT2G37970	1	0
AT2G38040	0	0
AT2G38120	0	0
AT2G38400	0	1
AT2G38410	0	0
AT2G38465	1	0
AT2G38470	0	0
AT2G38480	0	0
AT2G38640	1	0
AT2G38670	0	0
AT2G38710	0	0
AT2G38730	0	0
AT2G38740	0	0
AT2G38760	0	0
AT2G38780	0	0
AT2G38800	0	0
AT2G38820	1	0
AT2G38840	0	0
AT2G38860	1	0
AT2G39010	1	0

AT2G39450	1	0
AT2G39460	0	0
AT2G39470	1	0
AT2G39580	0	0
AT2G39650	0	1
AT2G39730	1	0
AT2G39750	0	0
AT2G39900	1	1
AT2G39920	1	0
AT2G39930	0	0
AT2G40000	0	0
AT2G40080	1	0
AT2G40100	1	0
AT2G40150	1	0
AT2G40230	0	0
AT2G40400	1	0
AT2G40420	1	0
AT2G40435	1	0
AT2G40460	1	0
AT2G40550	0	0
AT2G40840	1	0
AT2G40890	1	0
AT2G41000	0	0
AT2G41010	0	0
AT2G41040	1	0
AT2G41090	0	0
AT2G41120	1	1
AT2G41250	1	1
AT2G41290	1	0
AT2G41330	1	1
AT2G41500	0	0
AT2G41560	1	0
AT2G41620	0	0
AT2G41710	0	0
AT2G41760	1	0
AT2G41870	1	1
AT2G41880	0	0
AT2G41990	0	0
AT2G42030	0	0
AT2G42040	0	0
AT2G42200	0	0
AT2G42320	1	0
AT2G42330	0	0
AT2G42380	0	0
AT2G42400	0	0
AT2G42580	0	0
AT2G42590	0	0
AT2G42600	1	0
AT2G42620	0	0
AT2G42670	0	0

AT2G42740	0	0
AT2G42750	1	0
AT2G42770	0	0
AT2G42780	1	0
AT2G42790	0	0
AT2G42870	0	0
AT2G42890	0	0
AT2G42910	1	0
AT2G42950	0	0
AT2G43010	1	0
AT2G43090	0	0
AT2G43100	0	0
AT2G43210	0	0
AT2G43400	0	0
AT2G43530	1	0
AT2G43550	1	0
AT2G43710	1	0
AT2G43790	0	0
AT2G43820	0	0
AT2G44050	0	0
AT2G44150	0	0
AT2G44180	0	0
AT2G44300	0	0
AT2G44430	0	0
AT2G44490	1	0
AT2G44500	1	0
AT2G44670	1	0
AT2G44740	1	0
AT2G44860	1	0
AT2G44900	0	0
AT2G45050	0	0
AT2G45070	0	0
AT2G45140	0	0
AT2G45440	1	0
AT2G45620	0	0
AT2G45670	0	0
AT2G45720	0	0
AT2G45790	0	0
AT2G45910	0	0
AT2G45990	0	0
AT2G46220	0	0
AT2G46240	0	0
AT2G46260	0	0
AT2G46330	0	0
AT2G46340	1	0
AT2G46420	0	0
AT2G46450	1	1
AT2G46490	1	0
AT2G46550	1	0
AT2G46640	1	0

AT2G46650	0	0
AT2G46660	0	0
AT2G46710	1	0
AT2G46735	1	0
AT2G46830	1	1
AT2G46900	0	0
AT2G47010	1	0
AT2G47390	1	0
AT2G47450	0	0
AT2G47470	0	0
AT2G47490	1	1
AT2G47610	0	0
AT2G47790	0	0
AT2G47800	0	0
AT2G47840	0	0
AT2G47890	1	0
AT2G47960	0	0
AT2G48020	1	0
AT2G48030	0	0
AT2G48100	0	0
AT3G01060	1	0
AT3G01120	0	0
AT3G01140	0	0
AT3G01180	1	0
AT3G01210	1	0
AT3G01280	0	0
AT3G01310	1	0
AT3G01320	0	0
AT3G01400	0	0
AT3G01440	1	0
AT3G01490	1	0
AT3G01540	0	0
AT3G01550	1	1
AT3G01660	1	0
AT3G01690	1	0
AT3G02130	0	0
AT3G02150	0	0
AT3G02170	1	0
AT3G02340	0	0
AT3G02380	1	1
AT3G02390	0	0
AT3G02460	0	0
AT3G02540	0	0
AT3G02630	1	0
AT3G02660	1	0
AT3G02750	0	0
AT3G02820	0	0
AT3G02830	1	0
AT3G02870	0	0
AT3G02900	0	0

AT3G03020	0	0
AT3G03150	0	0
AT3G03190	0	0
AT3G03380	0	0
AT3G03770	1	0
AT3G03880	0	0
AT3G03960	0	0
AT3G03980	1	0
AT3G04120	1	0
AT3G04400	0	0
AT3G04520	1	0
AT3G04740	0	0
AT3G04770	0	0
AT3G04790	1	0
AT3G04830	1	0
AT3G04870	0	0
AT3G04880	0	0
AT3G04910	1	0
AT3G05120	0	0
AT3G05130	1	0
AT3G05180	0	0
AT3G05340	0	0
AT3G05640	1	0
AT3G05680	0	0
AT3G05810	0	0
AT3G05880	1	0
AT3G05900	1	0
AT3G05940	0	0
AT3G06035	1	0
AT3G06060	0	0
AT3G06070	1	0
AT3G06080	1	0
AT3G06170	0	0
AT3G06330	1	0
AT3G06340	0	0
AT3G06350	1	0
AT3G06440	0	0
AT3G06500	1	0
AT3G06510	1	0
AT3G06700	0	0
AT3G06750	0	0
AT3G06840	0	0
AT3G06850	0	0
AT3G07010	1	0
AT3G07020	0	0
AT3G07090	0	0
AT3G07170	0	0
AT3G07274	0	0
AT3G07350	1	0
AT3G07470	0	0

AT3G07590	0	0
AT3G07630	1	0
AT3G07640	1	1
AT3G07700	1	1
AT3G07720	1	0
AT3G07780	0	0
AT3G07930	0	0
AT3G08030	0	0
AT3G08510	0	0
AT3G08590	0	0
AT3G08640	0	0
AT3G08730	1	0
AT3G08760	1	0
AT3G08770	0	0
AT3G08930	0	0
AT3G08940	1	0
AT3G09030	0	0
AT3G09250	0	0
AT3G09310	0	0
AT3G09320	0	0
AT3G09390	1	0
AT3G09480	0	0
AT3G09540	0	0
AT3G09600	1	1
AT3G09770	0	0
AT3G09780	0	0
AT3G09820	0	0
AT3G09850	0	0
AT3G09880	0	0
AT3G09890	0	0
AT3G09920	0	0
AT3G09970	0	0
AT3G10020	1	0
AT3G10030	0	0
AT3G10230	1	0
AT3G10300	0	0
AT3G10410	1	0
AT3G10420	1	0
AT3G10520	1	0
AT3G10525	0	0
AT3G10550	0	0
AT3G10610	1	0
AT3G10720	0	0
AT3G10740	1	0
AT3G10760	1	0
AT3G10770	0	0
AT3G10800	0	0
AT3G10840	1	0
AT3G10940	0	0
AT3G11020	1	0

AT3G11100	0	0
AT3G11110	0	0
AT3G11210	0	0
AT3G11280	1	0
AT3G11510	1	0
AT3G11560	0	0
AT3G11600	1	0
AT3G11670	1	0
AT3G11690	1	0
AT3G11700	1	0
AT3G11760	0	0
AT3G11850	0	0
AT3G11900	1	1
AT3G11945	0	0
AT3G12020	0	0
AT3G12110	1	0
AT3G12290	1	0
AT3G12320	1	1
AT3G12370	0	0
AT3G12390	0	0
AT3G12570	0	0
AT3G12620	0	0
AT3G12685	0	0
AT3G12700	0	0
AT3G12760	0	0
AT3G12800	0	0
AT3G12920	0	0
AT3G12930	0	0
AT3G12940	0	0
AT3G12980	0	0
AT3G13040	1	0
AT3G13110	0	0
AT3G13230	1	0
AT3G13450	0	0
AT3G13560	0	0
AT3G13650	0	0
AT3G13690	1	0
AT3G13750	0	0
AT3G13790	0	0
AT3G13860	1	0
AT3G14010	0	0
AT3G14050	0	0
AT3G14075	0	0
AT3G14090	1	0
AT3G14180	0	0
AT3G14190	0	0
AT3G14210	1	0
AT3G14600	0	0
AT3G14750	0	0
AT3G14770	0	0

AT3G14910	0	0
AT3G14990	0	0
AT3G15040	0	0
AT3G15070	0	0
AT3G15180	1	0
AT3G15352	0	0
AT3G15450	1	0
AT3G15460	0	0
AT3G15510	0	0
AT3G15530	0	0
AT3G15540	1	0
AT3G15610	0	0
AT3G15630	1	0
AT3G15720	0	0
AT3G15840	1	0
AT3G15850	0	1
AT3G16120	0	0
AT3G16140	1	0
AT3G16150	1	0
AT3G16175	1	0
AT3G16180	0	0
AT3G16250	1	0
AT3G16520	1	0
AT3G16560	0	0
AT3G16750	0	0
AT3G16850	0	0
AT3G16857	0	0
AT3G16860	0	0
AT3G16910	1	0
AT3G17020	1	0
AT3G17040	1	1
AT3G17100	1	0
AT3G17120	0	0
AT3G17130	1	0
AT3G17390	0	0
AT3G17510	1	0
AT3G17609	1	1
AT3G17800	0	0
AT3G17820	0	0
AT3G17930	0	0
AT3G18035	0	0
AT3G18050	1	0
AT3G18060	0	0
AT3G18080	0	0
AT3G18130	1	0
AT3G18200	0	0
AT3G18420	1	0
AT3G18490	1	0
AT3G18710	0	0
AT3G18750	1	0

AT3G18760	0	0
AT3G18800	1	0
AT3G18830	0	0
AT3G18980	1	0
AT3G19170	0	0
AT3G19190	0	0
AT3G19380	0	0
AT3G19450	1	0
AT3G19480	0	0
AT3G19553	1	0
AT3G19720	1	0
AT3G19790	0	0
AT3G19930	0	0
AT3G19970	0	0
AT3G20240	0	0
AT3G20250	0	0
AT3G20270	0	0
AT3G20310	1	0
AT3G20330	0	0
AT3G20340	0	0
AT3G20410	0	0
AT3G20430	0	0
AT3G20500	1	0
AT3G20670	0	0
AT3G20770	1	0
AT3G20800	0	0
AT3G20810	1	1
AT3G20870	0	0
AT3G21060	0	0
AT3G21090	0	0
AT3G21110	1	0
AT3G21175	0	0
AT3G21190	1	0
AT3G21230	0	0
AT3G21240	0	0
AT3G21250	1	0
AT3G21390	0	0
AT3G21560	1	0
AT3G21650	0	0
AT3G21670	1	0
AT3G21690	1	0
AT3G21750	1	0
AT3G21760	1	0
AT3G21870	1	0
AT3G21890	1	0
AT3G22104	0	0
AT3G22110	0	0
AT3G22200	1	0
AT3G22230	0	0
AT3G22300	1	0

AT3G22420	0	0
AT3G22430	0	0
AT3G22460	0	0
AT3G22530	1	0
AT3G22540	0	0
AT3G22550	1	0
AT3G22630	0	0
AT3G22890	0	0
AT3G22960	1	0
AT3G23080	1	0
AT3G23210	1	0
AT3G23400	0	0
AT3G23410	0	0
AT3G23540	0	0
AT3G23570	0	0
AT3G23610	0	0
AT3G23640	1	0
AT3G23710	0	0
AT3G23810	1	0
AT3G23840	0	0
AT3G23920	1	0
AT3G23990	1	0
AT3G24150	0	0
AT3G24170	1	0
AT3G24190	1	1
AT3G24315	0	0
AT3G24430	1	0
AT3G24460	1	0
AT3G24590	0	0
AT3G25150	0	0
AT3G25220	0	0
AT3G25290	0	0
AT3G25480	0	0
AT3G25530	0	0
AT3G25545	1	0
AT3G25730	0	0
AT3G25740	0	0
AT3G25840	1	0
AT3G25910	0	0
AT3G26000	0	0
AT3G26030	0	0
AT3G26290	1	0
AT3G26310	0	0
AT3G26570	1	0
AT3G26580	1	0
AT3G26590	0	0
AT3G26740	1	0
AT3G26932	0	0
AT3G26980	0	0
AT3G27050	1	0

AT3G27060	0	0
AT3G27170	1	1
AT3G27260	0	0
AT3G27280	0	0
AT3G27300	0	0
AT3G27350	1	0
AT3G27380	0	0
AT3G27530	0	0
AT3G27560	0	0
AT3G27670	0	0
AT3G27690	1	1
AT3G27750	0	0
AT3G27890	0	0
AT3G27925	1	0
AT3G28040	0	0
AT3G28180	1	0
AT3G28220	0	0
AT3G28270	1	0
AT3G28540	0	0
AT3G28690	0	0
AT3G28850	1	0
AT3G28860	0	0
AT3G28900	1	0
AT3G29090	0	0
AT3G29320	1	0
AT3G29370	0	0
AT3G29760	1	0
AT3G32940	0	0
AT3G33520	0	0
AT3G42800	0	0
AT3G43600	0	0
AT3G43670	0	0
AT3G43720	0	0
AT3G43790	0	0
AT3G43800	0	0
AT3G44100	0	0
AT3G44320	1	0
AT3G44380	0	0
AT3G44450	1	0
AT3G44630	1	0
AT3G44720	0	0
AT3G44820	0	0
AT3G44970	1	0
AT3G44990	0	0
AT3G45010	1	0
AT3G45040	0	0
AT3G45050	1	0
AT3G45140	1	0
AT3G45210	0	0
AT3G45260	0	0

AT3G45300	1	0
AT3G45310	0	0
AT3G45780	1	0
AT3G45970	0	0
AT3G46030	0	0
AT3G46040	0	0
AT3G46380	0	0
AT3G46780	1	0
AT3G46940	0	0
AT3G46970	1	0
AT3G47160	1	0
AT3G47340	1	0
AT3G47370	0	0
AT3G47420	1	0
AT3G47430	1	0
AT3G47520	0	0
AT3G47550	1	0
AT3G47610	0	0
AT3G47620	0	0
AT3G47800	0	0
AT3G47833	0	0
AT3G47860	1	0
AT3G47990	0	0
AT3G48000	0	0
AT3G48460	1	1
AT3G48530	1	0
AT3G48610	1	0
AT3G48680	0	0
AT3G48700	0	0
AT3G48930	0	0
AT3G49000	0	0
AT3G49160	0	0
AT3G49220	0	0
AT3G49490	0	0
AT3G49650	0	0
AT3G49720	0	0
AT3G50060	0	0
AT3G50210	0	0
AT3G50240	0	0
AT3G50500	1	0
AT3G50560	0	0
AT3G50670	0	0
AT3G50685	0	0
AT3G50700	1	0
AT3G50750	1	0
AT3G50790	0	0
AT3G51110	1	0
AT3G51120	0	0
AT3G51160	0	0
AT3G51240	1	0

AT3G51290	0	0
AT3G51370	0	0
AT3G51390	0	0
AT3G51530	0	0
AT3G51550	1	0
AT3G51660	0	0
AT3G51770	0	0
AT3G51820	1	0
AT3G51840	1	0
AT3G51850	0	0
AT3G51870	1	0
AT3G51895	1	0
AT3G52060	0	0
AT3G52070	0	0
AT3G52180	1	0
AT3G52230	0	0
AT3G52240	0	0
AT3G52340	1	1
AT3G52360	1	1
AT3G52370	0	0
AT3G52390	0	0
AT3G52470	1	0
AT3G52590	0	0
AT3G52610	0	0
AT3G52720	0	0
AT3G52740	1	0
AT3G52800	0	0
AT3G52840	1	0
AT3G52880	0	0
AT3G52930	0	0
AT3G52940	0	0
AT3G52950	0	0
AT3G52960	1	0
AT3G53030	1	0
AT3G53130	1	0
AT3G53260	1	0
AT3G53430	0	0
AT3G53540	0	0
AT3G53570	0	0
AT3G53800	1	0
AT3G53880	0	0
AT3G53890	0	0
AT3G54050	0	0
AT3G54120	0	1
AT3G54360	0	0
AT3G54380	0	0
AT3G54500	1	1
AT3G54660	0	0
AT3G54680	0	0
AT3G54720	0	0

AT3G54840	0	0
AT3G54890	1	1
AT3G54900	1	0
AT3G54920	0	0
AT3G54950	0	0
AT3G54980	0	0
AT3G55040	1	0
AT3G55120	1	0
AT3G55280	0	0
AT3G55430	0	0
AT3G55450	1	0
AT3G55460	1	0
AT3G55760	1	0
AT3G55800	1	0
AT3G55960	1	0
AT3G56070	0	0
AT3G56080	1	0
AT3G56160	0	0
AT3G56200	1	0
AT3G56260	0	0
AT3G56290	1	0
AT3G56340	0	0
AT3G56370	1	0
AT3G56490	0	0
AT3G56590	0	0
AT3G56690	1	0
AT3G56720	0	0
AT3G56880	0	0
AT3G56940	1	0
AT3G56950	0	0
AT3G57010	0	0
AT3G57020	1	0
AT3G57030	0	0
AT3G57040	1	0
AT3G57070	0	0
AT3G57170	0	0
AT3G57290	1	0
AT3G57300	0	0
AT3G57390	0	0
AT3G57410	0	0
AT3G57480	0	0
AT3G57490	1	0
AT3G57880	1	0
AT3G58120	1	0
AT3G58570	1	0
AT3G58600	0	0
AT3G58620	1	0
AT3G58680	1	0
AT3G58700	1	0
AT3G58750	1	0

AT3G58990	1	0
AT3G59060	1	0
AT3G59080	0	0
AT3G59110	0	0
AT3G59140	0	0
AT3G59290	0	0
AT3G59350	1	0
AT3G59400	1	0
AT3G59540	0	0
AT3G59650	0	0
AT3G59690	0	0
AT3G59900	0	0
AT3G59940	1	0
AT3G60030	0	0
AT3G60080	1	0
AT3G60130	0	0
AT3G60220	0	0
AT3G60245	0	0
AT3G60390	0	0
AT3G60440	0	0
AT3G60530	1	0
AT3G60770	0	0
AT3G61010	0	0
AT3G61210	0	0
AT3G61220	1	0
AT3G61260	1	0
AT3G61570	1	0
AT3G61580	1	0
AT3G61600	0	0
AT3G61770	0	0
AT3G61890	1	0
AT3G62010	1	0
AT3G62130	1	0
AT3G62150	0	0
AT3G62410	1	0
AT3G62550	1	0
AT3G62580	0	0
AT3G62660	0	0
AT3G62770	0	0
AT3G62810	0	0
AT3G62860	0	0
AT3G62870	0	0
AT3G62910	1	0
AT3G63120	1	0
AT3G63140	0	0
AT3G63160	1	0
AT3G63210	0	0
AT4G00050	1	1
AT4G00300	0	0
AT4G00490	1	0

AT4G00810	0	0
AT4G00840	0	0
AT4G00860	0	0
AT4G00900	1	0
AT4G00950	0	0
AT4G01050	0	0
AT4G01070	1	1
AT4G01080	1	0
AT4G01120	0	0
AT4G01130	1	0
AT4G01250	1	0
AT4G01330	1	0
AT4G01410	0	0
AT4G01480	0	0
AT4G01550	0	0
AT4G01660	0	0
AT4G01680	0	0
AT4G01700	0	0
AT4G02130	0	0
AT4G02200	0	0
AT4G02370	1	0
AT4G02420	1	0
AT4G02440	0	0
AT4G02480	0	0
AT4G02500	0	0
AT4G02530	0	0
AT4G02540	0	0
AT4G02850	0	0
AT4G02880	0	0
AT4G02890	1	0
AT4G02920	1	0
AT4G03020	0	0
AT4G03030	0	0
AT4G03060	0	0
AT4G03190	0	0
AT4G03210	0	0
AT4G03390	0	0
AT4G03510	1	0
AT4G03560	1	0
AT4G04210	0	0
AT4G04330	1	0
AT4G04340	1	0
AT4G04350	0	0
AT4G04610	0	0
AT4G04630	0	0
AT4G04850	1	0
AT4G04870	0	0
AT4G04955	0	1
AT4G05070	1	0
AT4G05150	1	0

AT4G05180	1	0
AT4G05330	0	0
AT4G05420	0	0
AT4G05450	0	0
AT4G08110	0	0
AT4G08240	0	0
AT4G08590	1	0
AT4G08920	1	0
AT4G08960	0	0
AT4G08980	1	0
AT4G09020	1	0
AT4G09030	0	0
AT4G09140	0	0
AT4G09160	0	0
AT4G09320	0	0
AT4G09350	1	0
AT4G09620	0	0
AT4G09670	0	0
AT4G09760	1	0
AT4G09800	0	0
AT4G09970	0	0
AT4G10040	0	0
AT4G10120	1	1
AT4G10170	0	0
AT4G10300	1	0
AT4G10450	0	0
AT4G10480	0	0
AT4G10760	0	0
AT4G10770	1	0
AT4G10840	0	0
AT4G10925	0	0
AT4G10970	0	0
AT4G11410	0	0
AT4G11570	1	0
AT4G11600	1	0
AT4G11740	0	0
AT4G12000	1	0
AT4G12030	0	0
AT4G12040	0	0
AT4G12060	0	0
AT4G12070	1	0
AT4G12390	1	0
AT4G12420	1	0
AT4G12570	0	0
AT4G12600	1	0
AT4G12690	0	0
AT4G12730	0	0
AT4G12750	0	0
AT4G12800	1	0
AT4G12830	1	0

AT4G12880	1	0
AT4G12980	1	0
AT4G13030	0	0
AT4G13050	1	0
AT4G13170	0	0
AT4G13180	0	0
AT4G13235	0	0
AT4G13250	1	0
AT4G13510	1	0
AT4G13530	0	0
AT4G13640	1	0
AT4G13770	1	0
AT4G13850	1	0
AT4G14020	1	0
AT4G14160	0	0
AT4G14230	1	0
AT4G14240	0	0
AT4G14270	1	0
AT4G14400	0	0
AT4G14420	0	0
AT4G14440	0	0
AT4G14500	0	0
AT4G14550	1	1
AT4G14660	1	0
AT4G14680	0	0
AT4G14720	0	0
AT4G14910	0	0
AT4G14960	0	0
AT4G14990	0	0
AT4G15010	0	0
AT4G15430	1	0
AT4G15440	1	0
AT4G15530	1	0
AT4G15550	0	0
AT4G15560	1	0
AT4G15620	0	0
AT4G16060	1	0
AT4G16130	0	0
AT4G16140	1	0
AT4G16146	1	0
AT4G16170	0	0
AT4G16190	1	0
AT4G16265	0	0
AT4G16320	0	0
AT4G16360	1	0
AT4G16370	0	0
AT4G16440	0	0
AT4G16447	0	0
AT4G16480	0	0
AT4G16490	1	0

AT4G16690	1	0
AT4G16720	0	0
AT4G16780	1	0
AT4G16860	0	0
AT4G16870	0	0
AT4G16950	1	0
AT4G16980	1	0
AT4G16990	1	0
AT4G17030	0	0
AT4G17080	0	0
AT4G17090	1	0
AT4G17100	1	0
AT4G17140	0	0
AT4G17150	1	0
AT4G17170	0	0
AT4G17230	0	0
AT4G17390	0	0
AT4G17460	1	0
AT4G17470	0	0
AT4G17530	1	0
AT4G17650	0	0
AT4G17740	0	0
AT4G17770	0	0
AT4G17840	1	1
AT4G17900	0	0
AT4G18140	0	0
AT4G18160	0	0
AT4G18520	0	0
AT4G18530	0	0
AT4G18710	0	0
AT4G18800	0	0
AT4G18810	1	1
AT4G18880	0	0
AT4G18930	1	0
AT4G19120	1	0
AT4G19160	0	0
AT4G19170	1	0
AT4G19380	0	0
AT4G19410	1	0
AT4G19450	0	0
AT4G19710	1	0
AT4G19830	0	0
AT4G19860	0	0
AT4G20070	0	0
AT4G20130	0	0
AT4G20170	1	0
AT4G20260	0	0
AT4G20280	0	0
AT4G20440	0	0
AT4G21090	0	0

AT4G21180	0	0
AT4G21210	1	0
AT4G21350	1	0
AT4G21400	0	0
AT4G21470	0	0
AT4G21710	0	0
AT4G21750	0	0
AT4G21760	0	0
AT4G21940	1	0
AT4G21990	0	0
AT4G22540	0	0
AT4G22570	1	0
AT4G22610	0	0
AT4G22730	0	0
AT4G22756	0	0
AT4G22780	0	0
AT4G22890	0	0
AT4G22930	0	0
AT4G23040	0	0
AT4G23180	1	0
AT4G23270	1	0
AT4G23290	1	0
AT4G23600	0	0
AT4G23620	0	0
AT4G23820	1	0
AT4G23840	1	0
AT4G23900	0	0
AT4G24020	0	0
AT4G24040	0	0
AT4G24100	0	0
AT4G24130	1	0
AT4G24220	0	0
AT4G24290	0	0
AT4G24460	0	0
AT4G24520	0	0
AT4G24660	0	0
AT4G24690	0	0
AT4G24700	1	1
AT4G24800	1	0
AT4G24810	0	0
AT4G24940	0	0
AT4G24960	0	0
AT4G25170	0	0
AT4G25260	0	0
AT4G25570	1	0
AT4G25640	1	0
AT4G25650	0	0
AT4G25700	1	1
AT4G25740	1	0
AT4G25830	1	0

AT4G25940	0	0
AT4G25960	0	0
AT4G25970	1	0
AT4G26130	1	0
AT4G26210	1	0
AT4G26400	1	0
AT4G26500	0	0
AT4G26670	1	0
AT4G26690	0	0
AT4G26700	1	0
AT4G26790	1	0
AT4G26850	1	1
AT4G26860	1	0
AT4G27030	0	0
AT4G27050	0	0
AT4G27080	0	0
AT4G27320	1	0
AT4G27350	0	0
AT4G27430	0	0
AT4G27440	1	0
AT4G27470	0	0
AT4G27520	0	0
AT4G27880	0	0
AT4G27940	0	0
AT4G28025	0	0
AT4G28040	0	0
AT4G28250	1	0
AT4G28260	0	0
AT4G28290	0	0
AT4G28300	0	0
AT4G28440	0	0
AT4G28480	1	0
AT4G28540	0	0
AT4G28660	1	0
AT4G28720	0	0
AT4G28740	0	0
AT4G29010	0	0
AT4G29190	1	0
AT4G29350	0	0
AT4G29380	0	0
AT4G29410	0	0
AT4G29480	0	0
AT4G29590	1	0
AT4G29700	0	0
AT4G29810	0	0
AT4G29840	1	0
AT4G29950	1	0
AT4G30020	0	0
AT4G30270	0	0
AT4G30350	0	0

AT4G30360	0	0
AT4G30470	1	1
AT4G30480	0	0
AT4G30530	0	0
AT4G30630	0	0
AT4G30660	1	0
AT4G30690	1	0
AT4G30790	0	0
AT4G30800	0	0
AT4G30900	0	0
AT4G30940	0	0
AT4G30960	1	0
AT4G31040	1	0
AT4G31050	1	0
AT4G31080	0	0
AT4G31290	1	0
AT4G31430	0	0
AT4G31500	1	0
AT4G31540	0	0
AT4G31590	1	0
AT4G31810	1	0
AT4G31870	1	0
AT4G31985	0	0
AT4G32020	1	0
AT4G32060	0	0
AT4G32190	1	0
AT4G32350	0	0
AT4G32760	0	0
AT4G32770	1	1
AT4G32850	0	0
AT4G32890	0	0
AT4G32915	0	0
AT4G33010	1	0
AT4G33040	1	0
AT4G33090	1	0
AT4G33140	1	0
AT4G33150	0	0
AT4G33210	0	0
AT4G33300	1	0
AT4G33380	0	0
AT4G33490	1	1
AT4G33520	1	0
AT4G33580	0	0
AT4G33700	1	0
AT4G33740	0	0
AT4G33770	0	0
AT4G33980	1	0
AT4G34030	0	0
AT4G34090	0	0
AT4G34100	0	0

AT4G34150	1	0
AT4G34190	0	0
AT4G34200	0	0
AT4G34230	1	0
AT4G34240	0	0
AT4G34290	0	0
AT4G34350	1	0
AT4G34450	1	0
AT4G34610	1	0
AT4G34630	1	0
AT4G34650	0	0
AT4G34700	0	0
AT4G34730	0	0
AT4G34760	1	0
AT4G34870	0	0
AT4G35020	0	0
AT4G35090	1	0
AT4G35250	1	0
AT4G35300	0	0
AT4G35320	0	0
AT4G35470	1	0
AT4G35480	0	0
AT4G35630	0	0
AT4G35770	1	0
AT4G35790	0	0
AT4G35830	0	0
AT4G35840	0	0
AT4G35870	0	0
AT4G35950	0	0
AT4G36040	1	0
AT4G36050	0	0
AT4G36360	1	0
AT4G36400	0	0
AT4G36540	1	0
AT4G36640	0	0
AT4G36660	0	0
AT4G36670	0	0
AT4G36730	0	0
AT4G36760	0	0
AT4G36830	0	0
AT4G36930	0	0
AT4G37110	0	0
AT4G37180	0	0
AT4G37250	1	0
AT4G37260	0	0
AT4G37310	0	0
AT4G37330	0	0
AT4G37460	1	0
AT4G37550	1	0
AT4G37560	0	0

AT4G37660	0	0
AT4G37760	1	1
AT4G37790	0	0
AT4G37800	0	0
AT4G37870	1	0
AT4G37890	0	0
AT4G37910	1	0
AT4G38100	1	0
AT4G38160	1	0
AT4G38470	1	0
AT4G38580	1	0
AT4G38620	0	0
AT4G38900	0	0
AT4G38960	1	0
AT4G38970	1	0
AT4G39030	0	0
AT4G39090	0	0
AT4G39150	0	0
AT4G39210	1	0
AT4G39270	0	0
AT4G39330	1	0
AT4G39400	0	0
AT4G39450	0	0
AT4G39510	1	0
AT4G39710	0	0
AT4G39730	0	0
AT4G39800	1	0
AT4G39840	1	0
AT4G39910	0	0
AT4G39940	1	0
AT4G39980	1	0
AT4G39990	0	0
AT4G40050	0	0
AT5G01015	0	0
AT5G01075	1	0
AT5G01210	1	0
AT5G01260	1	0
AT5G01410	1	1
AT5G01460	1	0
AT5G01520	1	0
AT5G01530	1	0
AT5G01720	0	0
AT5G01730	0	0
AT5G01750	1	0
AT5G01810	0	0
AT5G01820	1	0
AT5G01950	0	0
AT5G02020	0	0
AT5G02050	1	0
AT5G02100	0	0

AT5G02120	1	0
AT5G02150	1	0
AT5G02160	1	0
AT5G02180	1	0
AT5G02230	1	0
AT5G02240	1	0
AT5G02280	1	0
AT5G02290	0	0
AT5G02310	0	0
AT5G02440	0	0
AT5G02540	0	0
AT5G02710	1	0
AT5G02810	1	1
AT5G02830	1	0
AT5G02840	1	0
AT5G02860	1	0
AT5G02940	0	0
AT5G03050	0	0
AT5G03120	0	0
AT5G03130	1	0
AT5G03150	0	0
AT5G03200	0	0
AT5G03260	0	0
AT5G03290	0	0
AT5G03300	0	0
AT5G03350	0	0
AT5G03470	1	0
AT5G03490	0	0
AT5G03555	1	1
AT5G03760	1	0
AT5G03850	0	0
AT5G03905	0	0
AT5G04040	1	0
AT5G04110	0	0
AT5G04190	0	0
AT5G04220	0	0
AT5G04250	0	0
AT5G04480	0	0
AT5G04490	1	0
AT5G04530	1	0
AT5G04560	0	0
AT5G04770	0	0
AT5G04820	1	0
AT5G04830	0	0
AT5G04900	0	0
AT5G04950	0	0
AT5G05170	0	0
AT5G05250	0	0
AT5G05270	1	0
AT5G05320	1	0

AT5G05410	1	0
AT5G05690	1	0
AT5G05870	0	0
AT5G05890	1	0
AT5G05930	0	0
AT5G05960	0	0
AT5G06060	0	0
AT5G06270	1	0
AT5G06530	1	0
AT5G06560	0	0
AT5G06690	1	0
AT5G06770	0	0
AT5G06790	0	0
AT5G06830	1	0
AT5G06910	0	0
AT5G06930	0	0
AT5G07000	1	0
AT5G07010	0	0
AT5G07020	0	0
AT5G07090	0	0
AT5G07100	0	0
AT5G07220	0	0
AT5G07240	0	0
AT5G07290	0	0
AT5G07320	0	0
AT5G07370	0	0
AT5G07440	1	0
AT5G07460	0	0
AT5G07690	1	0
AT5G08000	1	0
AT5G08050	0	0
AT5G08100	0	0
AT5G08180	0	0
AT5G08300	0	0
AT5G08330	1	0
AT5G08380	1	0
AT5G08400	1	0
AT5G08410	1	0
AT5G08520	0	0
AT5G08640	1	0
AT5G08650	0	1
AT5G09230	0	0
AT5G09270	0	0
AT5G09410	0	0
AT5G09520	0	0
AT5G09620	1	0
AT5G09650	0	0
AT5G09740	0	0
AT5G09810	0	0
AT5G09820	1	0

AT5G09870	1	1
AT5G09990	1	0
AT5G10070	0	0
AT5G10390	0	0
AT5G10400	0	0
AT5G10450	0	0
AT5G10470	1	0
AT5G10480	0	0
AT5G10540	0	0
AT5G10560	1	0
AT5G10695	1	0
AT5G10730	0	0
AT5G10740	0	0
AT5G10790	0	0
AT5G10860	1	0
AT5G10930	0	0
AT5G11060	0	0
AT5G11110	0	0
AT5G11160	0	0
AT5G11250	0	1
AT5G11260	0	1
AT5G11340	0	0
AT5G11420	1	0
AT5G11580	1	0
AT5G11610	0	0
AT5G11670	1	0
AT5G11710	0	0
AT5G11790	0	0
AT5G11840	1	0
AT5G11950	1	0
AT5G11960	0	0
AT5G12050	1	0
AT5G12140	0	0
AT5G12150	1	0
AT5G12170	0	0
AT5G12250	0	0
AT5G12430	0	0
AT5G12470	0	0
AT5G12840	1	0
AT5G13010	0	0
AT5G13090	1	0
AT5G13120	1	0
AT5G13180	1	0
AT5G13400	1	0
AT5G13550	0	0
AT5G13630	1	0
AT5G13710	0	0
AT5G13730	1	0
AT5G13750	0	0
AT5G13760	0	0

AT5G13770	0	0
AT5G13850	0	0
AT5G13930	1	0
AT5G13950	0	0
AT5G14030	0	0
AT5G14080	0	0
AT5G14170	0	0
AT5G14260	1	0
AT5G14270	1	0
AT5G14390	0	0
AT5G14530	1	0
AT5G14550	0	0
AT5G14640	1	0
AT5G14660	0	0
AT5G14700	1	0
AT5G14760	1	1
AT5G14880	1	0
AT5G14950	0	0
AT5G15020	1	0
AT5G15090	0	0
AT5G15200	0	0
AT5G15230	1	0
AT5G15280	0	0
AT5G15350	1	0
AT5G15450	1	0
AT5G15490	0	0
AT5G15540	0	0
AT5G15580	0	0
AT5G15600	0	0
AT5G15640	0	0
AT5G15680	0	0
AT5G15710	0	0
AT5G15740	1	0
AT5G15760	0	0
AT5G15770	0	0
AT5G15830	0	0
AT5G15850	1	0
AT5G15880	0	0
AT5G15910	1	0
AT5G15920	0	0
AT5G16030	1	0
AT5G16110	0	0
AT5G16130	0	0
AT5G16180	0	0
AT5G16200	0	0
AT5G16240	0	0
AT5G16260	0	0
AT5G16480	0	0
AT5G16560	0	0
AT5G16590	0	0

AT5G16630	0	0
AT5G16660	1	0
AT5G16820	0	0
AT5G16840	0	0
AT5G16970	1	0
AT5G17010	0	0
AT5G17050	1	0
AT5G17060	0	0
AT5G17170	0	0
AT5G17230	1	0
AT5G17280	0	0
AT5G17290	0	0
AT5G17300	1	1
AT5G17310	1	0
AT5G17560	1	0
AT5G17600	0	0
AT5G17640	0	0
AT5G17670	0	0
AT5G17690	0	0
AT5G17780	0	0
AT5G17840	0	0
AT5G17860	0	0
AT5G17990	1	0
AT5G18130	1	0
AT5G18140	0	0
AT5G18170	0	0
AT5G18525	0	0
AT5G18590	1	0
AT5G18650	0	0
AT5G18670	1	1
AT5G18680	1	0
AT5G18750	0	0
AT5G18760	1	0
AT5G18970	0	0
AT5G19010	0	0
AT5G19140	1	0
AT5G19220	0	0
AT5G19240	0	0
AT5G19330	0	0
AT5G19370	0	0
AT5G19440	0	0
AT5G19500	1	0
AT5G19660	0	0
AT5G19680	0	0
AT5G19850	1	1
AT5G19855	0	0
AT5G19875	1	0
AT5G20030	0	0
AT5G20060	0	0
AT5G20070	1	0

AT5G20080	0	0
AT5G20140	0	0
AT5G20160	0	0
AT5G20220	1	0
AT5G20250	1	0
AT5G20270	0	0
AT5G20350	0	0
AT5G20360	0	0
AT5G20380	1	0
AT5G20630	1	0
AT5G20840	0	0
AT5G20935	0	0
AT5G21100	1	0
AT5G21160	0	0
AT5G21170	1	0
AT5G22300	0	0
AT5G22310	1	0
AT5G22390	0	0
AT5G22460	1	0
AT5G22630	0	0
AT5G22710	0	0
AT5G22880	0	0
AT5G22920	1	0
AT5G22950	0	0
AT5G23060	1	0
AT5G23080	0	0
AT5G23130	0	0
AT5G23240	0	0
AT5G23250	0	0
AT5G23380	1	0
AT5G23390	0	0
AT5G23590	0	0
AT5G23610	0	0
AT5G23670	1	0
AT5G23730	1	0
AT5G23740	1	0
AT5G23750	1	0
AT5G23860	0	0
AT5G23870	0	0
AT5G23920	0	0
AT5G23940	0	0
AT5G24010	0	0
AT5G24120	1	0
AT5G24150	1	1
AT5G24320	0	0
AT5G24430	0	0
AT5G24470	0	0
AT5G24490	1	0
AT5G24520	0	0
AT5G24610	1	0

AT5G24620	0	0
AT5G24660	0	0
AT5G24810	1	0
AT5G24850	0	0
AT5G24870	0	0
AT5G24890	1	0
AT5G24930	1	0
AT5G25070	0	0
AT5G25210	1	0
AT5G25360	0	0
AT5G25460	1	0
AT5G25475	0	0
AT5G25890	0	0
AT5G26040	0	0
AT5G26200	0	0
AT5G26570	1	0
AT5G26720	0	0
AT5G26740	0	0
AT5G26790	1	0
AT5G27280	1	0
AT5G27320	1	0
AT5G27360	1	0
AT5G27380	1	0
AT5G27650	0	0
AT5G27760	0	0
AT5G27930	0	0
AT5G28770	1	0
AT5G29000	0	0
AT5G32440	0	0
AT5G33370	0	0
AT5G35180	0	0
AT5G35200	0	0
AT5G35460	1	0
AT5G35560	0	0
AT5G35620	0	0
AT5G35735	1	0
AT5G35740	0	0
AT5G35750	0	0
AT5G35970	1	0
AT5G36160	0	0
AT5G36220	0	0
AT5G37290	0	0
AT5G37360	1	0
AT5G37540	0	0
AT5G37680	0	0
AT5G37710	0	0
AT5G37780	0	0
AT5G37790	0	0
AT5G38020	0	0
AT5G38110	0	0

AT5G38140	0	0
AT5G38150	0	0
AT5G38380	0	0
AT5G38520	0	0
AT5G38530	0	0
AT5G38880	0	0
AT5G38890	0	0
AT5G38980	1	0
AT5G39080	1	0
AT5G39210	1	0
AT5G39350	0	0
AT5G39410	1	0
AT5G39590	0	0
AT5G39660	0	1
AT5G40170	1	0
AT5G40300	0	0
AT5G40380	1	0
AT5G40390	1	0
AT5G40450	0	0
AT5G40540	0	0
AT5G40550	0	0
AT5G40690	0	0
AT5G40870	0	0
AT5G41060	0	0
AT5G41330	0	0
AT5G41400	0	0
AT5G41460	1	0
AT5G41520	1	0
AT5G41650	0	0
AT5G41920	0	0
AT5G41940	1	0
AT5G42070	1	0
AT5G42080	0	0
AT5G42420	0	0
AT5G42620	0	0
AT5G42670	0	0
AT5G42720	0	0
AT5G42760	0	1
AT5G42810	1	0
AT5G42820	0	0
AT5G42860	1	0
AT5G42870	1	0
AT5G42900	1	0
AT5G42920	0	0
AT5G42990	0	0
AT5G43070	1	0
AT5G43150	0	0
AT5G43190	0	0
AT5G43440	1	0
AT5G43750	0	0

AT5G43790	0	0
AT5G43830	1	0
AT5G43870	0	0
AT5G43880	0	0
AT5G43930	0	0
AT5G44020	1	0
AT5G44050	0	0
AT5G44080	0	0
AT5G44090	0	0
AT5G44110	0	0
AT5G44130	0	0
AT5G44180	0	0
AT5G44190	1	0
AT5G44260	1	0
AT5G44400	0	0
AT5G44530	0	0
AT5G44650	1	0
AT5G44680	1	0
AT5G44730	0	0
AT5G44790	0	0
AT5G44860	0	0
AT5G45110	0	0
AT5G45170	0	0
AT5G45280	1	0
AT5G45360	0	0
AT5G45590	0	0
AT5G45710	0	0
AT5G45750	0	0
AT5G45800	0	0
AT5G45820	1	0
AT5G45950	1	0
AT5G46230	1	0
AT5G46295	0	0
AT5G46470	0	0
AT5G46630	1	0
AT5G46700	0	0
AT5G46710	1	0
AT5G46790	0	0
AT5G46800	1	0
AT5G47020	0	0
AT5G47200	0	0
AT5G47240	1	0
AT5G47320	1	0
AT5G47390	0	0
AT5G47500	0	0
AT5G47560	1	0
AT5G47580	0	0
AT5G47610	1	0
AT5G47650	0	0
AT5G47730	0	0

AT5G47870	0	0
AT5G47910	0	0
AT5G47930	0	0
AT5G47970	0	0
AT5G48000	0	0
AT5G48010	0	0
AT5G48030	0	0
AT5G48150	0	0
AT5G48250	1	0
AT5G48490	1	0
AT5G48540	1	0
AT5G48830	1	0
AT5G48870	0	0
AT5G48990	1	0
AT5G49230	0	0
AT5G49360	1	0
AT5G49480	1	0
AT5G49650	1	0
AT5G49700	1	0
AT5G49710	0	0
AT5G49720	0	0
AT5G49760	1	0
AT5G49820	0	0
AT5G49840	0	0
AT5G50000	0	0
AT5G50100	1	0
AT5G50110	0	0
AT5G50240	0	0
AT5G50250	0	0
AT5G50370	0	0
AT5G50380	0	0
AT5G50450	1	0
AT5G50460	0	0
AT5G50915	0	0
AT5G51040	1	0
AT5G51070	1	0
AT5G51110	1	1
AT5G51180	1	0
AT5G51460	1	0
AT5G51570	0	0
AT5G51720	0	0
AT5G51820	1	0
AT5G52030	0	0
AT5G52060	1	0
AT5G52320	0	0
AT5G52420	0	0
AT5G52570	1	1
AT5G52650	0	0
AT5G52780	1	0
AT5G52810	1	0

AT5G52900	1	0
AT5G53070	0	0
AT5G53150	0	0
AT5G53280	0	0
AT5G53370	1	1
AT5G53440	0	0
AT5G53490	1	0
AT5G53580	1	0
AT5G53750	0	0
AT5G53760	1	0
AT5G53970	1	1
AT5G54080	1	0
AT5G54110	1	0
AT5G54130	0	1
AT5G54190	0	0
AT5G54300	0	0
AT5G54310	0	0
AT5G54630	1	0
AT5G54680	0	0
AT5G54730	0	0
AT5G54930	1	0
AT5G54970	0	0
AT5G55050	0	0
AT5G55300	0	0
AT5G55380	1	0
AT5G55480	0	0
AT5G55600	0	0
AT5G55620	0	0
AT5G55700	0	0
AT5G55710	0	0
AT5G55910	0	0
AT5G55970	0	0
AT5G56100	0	0
AT5G56180	1	0
AT5G56190	0	0
AT5G56230	0	0
AT5G56750	0	0
AT5G56870	0	0
AT5G56900	0	0
AT5G57000	0	0
AT5G57080	0	0
AT5G57110	1	0
AT5G57340	1	0
AT5G57345	1	0
AT5G57360	0	0
AT5G57630	1	0
AT5G57655	1	0
AT5G57660	1	0
AT5G57830	1	0
AT5G57960	0	0

AT5G58120	1	0
AT5G58140	0	0
AT5G58150	0	0
AT5G58260	1	0
AT5G58290	0	0
AT5G58390	0	0
AT5G58430	0	0
AT5G58490	0	0
AT5G58710	0	0
AT5G58720	0	0
AT5G58760	0	0
AT5G58770	1	0
AT5G58787	0	0
AT5G58870	1	0
AT5G58900	0	0
AT5G59050	0	0
AT5G59080	1	0
AT5G59130	0	0
AT5G59420	1	0
AT5G59480	1	0
AT5G59500	0	0
AT5G59730	1	0
AT5G59780	1	0
AT5G59870	0	0
AT5G59910	0	0
AT5G60100	1	1
AT5G60670	0	0
AT5G60840	0	0
AT5G60850	1	0
AT5G60950	0	0
AT5G61010	1	0
AT5G61020	1	0
AT5G61030	0	0
AT5G61210	0	0
AT5G61380	1	0
AT5G61420	0	0
AT5G61450	0	0
AT5G61520	0	0
AT5G61580	1	0
AT5G61590	1	0
AT5G61600	0	0
AT5G61760	0	0
AT5G61820	0	0
AT5G61990	0	0
AT5G62030	0	0
AT5G62130	1	0
AT5G62140	0	0
AT5G62220	1	1
AT5G62350	1	0
AT5G62360	0	0

AT5G62430	1	1
AT5G62470	1	0
AT5G62530	0	0
AT5G62570	0	0
AT5G62610	0	0
AT5G62630	0	0
AT5G62840	1	0
AT5G62910	0	0
AT5G63190	1	0
AT5G63200	1	0
AT5G63220	0	0
AT5G63320	0	0
AT5G63370	0	0
AT5G63380	0	0
AT5G63400	0	0
AT5G63440	0	0
AT5G63510	0	0
AT5G63620	0	0
AT5G63650	0	0
AT5G63700	0	0
AT5G63780	1	1
AT5G63810	1	0
AT5G63860	1	0
AT5G63890	0	0
AT5G63980	1	0
AT5G64090	0	0
AT5G64330	1	0
AT5G64360	0	0
AT5G64460	0	0
AT5G64813	0	0
AT5G64840	1	0
AT5G64850	1	0
AT5G64860	1	0
AT5G64920	0	0
AT5G64930	0	0
AT5G64940	1	0
AT5G65110	0	0
AT5G65360	0	0
AT5G65380	1	0
AT5G65430	1	0
AT5G65480	0	0
AT5G65490	0	0
AT5G65540	0	0
AT5G65860	0	0
AT5G65870	1	0
AT5G65960	0	0
AT5G65990	1	0
AT5G66050	0	0
AT5G66070	1	0
AT5G66080	1	0

AT5G66160	0	0
AT5G66180	0	0
AT5G66460	0	0
AT5G66570	1	0
AT5G66880	1	0
AT5G67030	1	1
AT5G67150	0	0
AT5G67160	0	0
AT5G67330	0	0
AT5G67370	1	0
AT5G67440	0	0
AT5G67480	1	0
AT5G67600	0	0
Number in common	1164	109
Total in comparitor list	2885	109

Table S3. Comparison of phase module composition across species. The Jaccard similarity score, TP/(TP + FP + FN), was used to score the overlap between phase module compositions in Arabidopsis and Oryza sativa using orthologs identified as circadian in both. The, standard Deviation (STD) and mean of the distribution of Jaccard similarity scores on randomized module partitioning are displayed, as is the normalized Jaccard similarity score, that is, the number of standard deviations from the mean . Classes 1 and 2 correspond to subjective dawn and subjective dusk-phased genes respectively whereas classes 3 and 4 correspond to the middle of the subjective day and night respectively.

ID	STD	Mean	Jaccard	Normalized x STD
**class1**	0.00375	0.414	0.2716	61.011
**class2**	0.006995	0.020862	0.26574	35.007
**class3**	0.00178	0.085	0.1758	53.55
**class4**	0.00338	0.06331	0.1898	42.96
